# Supplementary material for: Tetrahelical structural family adopted by AGCGA-rich regulatory DNA regions
Source: Nat Commun. 2017 May 17;8:15355. doi: 10.1038/ncomms15355 (PMC5442326; doi:10.1038/ncomms15355)
Supplement: Supplementary Information — Supplementary Figures and Supplementary Tables [file ncomms15355-s1.pdf]

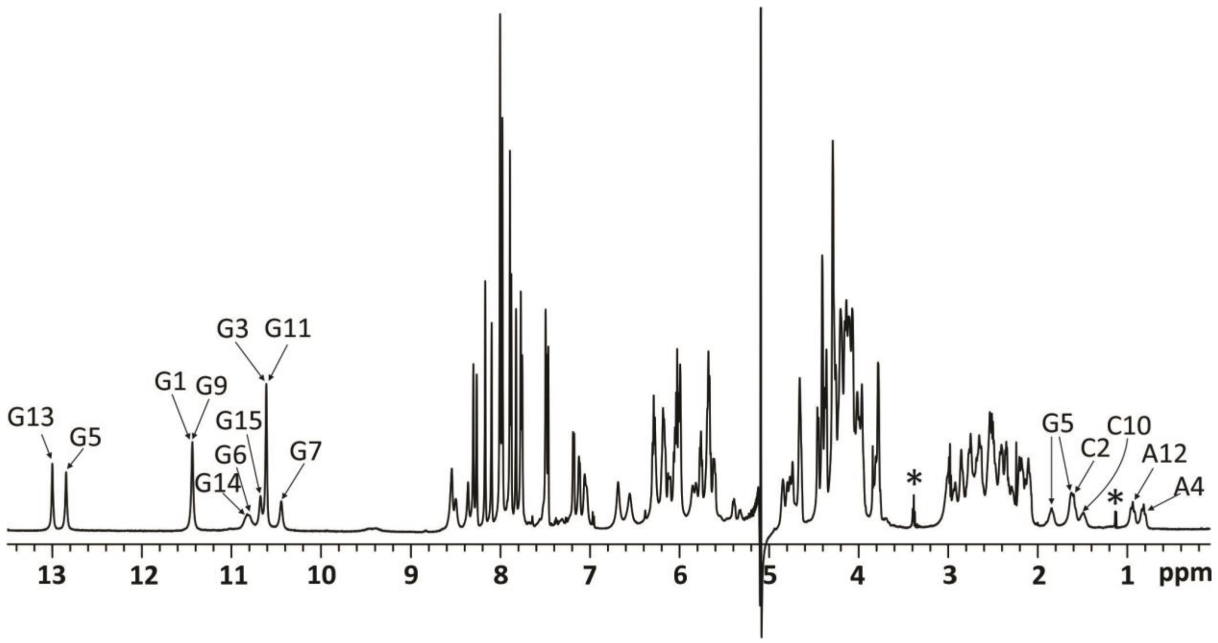

Supplementary Figure 1: 1D ^{1}H NMR spectrum of VK34 dimer. The assignment of imino protons of guanine residues and G5 H2”, G5 H2’, C2 H2’, C10 H2’, A12 H2’ and A4 H2’ protons is indicated. The two signals of unknown impurity are labelled with *. The spectrum was recorded at 0.4 mM oligonucleotide concentration per strand, 100 mM LiCl, pH 6.0 and 0 °C on an 800 MHz spectrometer.

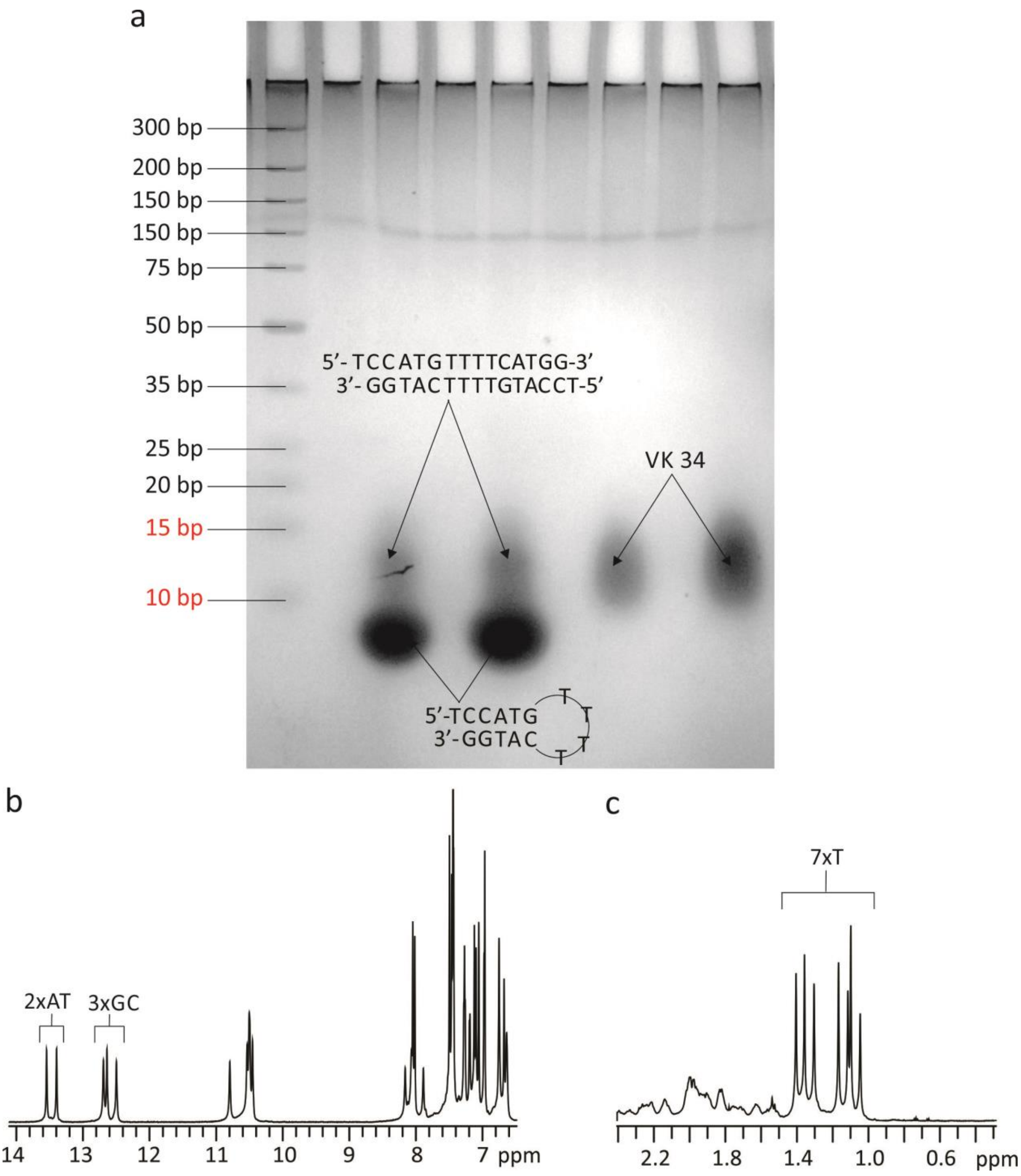

Supplementary Figure 2: Determination of the dimeric nature of VK34 by 10% native PAGE gel with 100 mM LiCl (a). Bands corresponding to hairpin and duplex used as control, VK34 dimer and DNA ladder with 300-10 base pairs are indicated. Each sample was applied twice. The 15 and 10 base pair bands are highlighted in red. (**b**) Imino, aromatic and **(c)** aliphatic regions of 1D ^{1}H NMR spectrum of d(TCCATGTTTTCATGG) with hairpin structure as the major species. Two signals that belong to the thymine imino protons involved in A-T pairs, three signals that belong to guanine H1 protons involved in G-C pairs and seven signals that belong to thymine CH_3 protons are indicated. The spectrum was recorded at 1.0 mM oligonucleotide concentration per strand, pH 6.0, 0 °C and 100 mM LiCl concentration on an 800 MHz spectrometer.

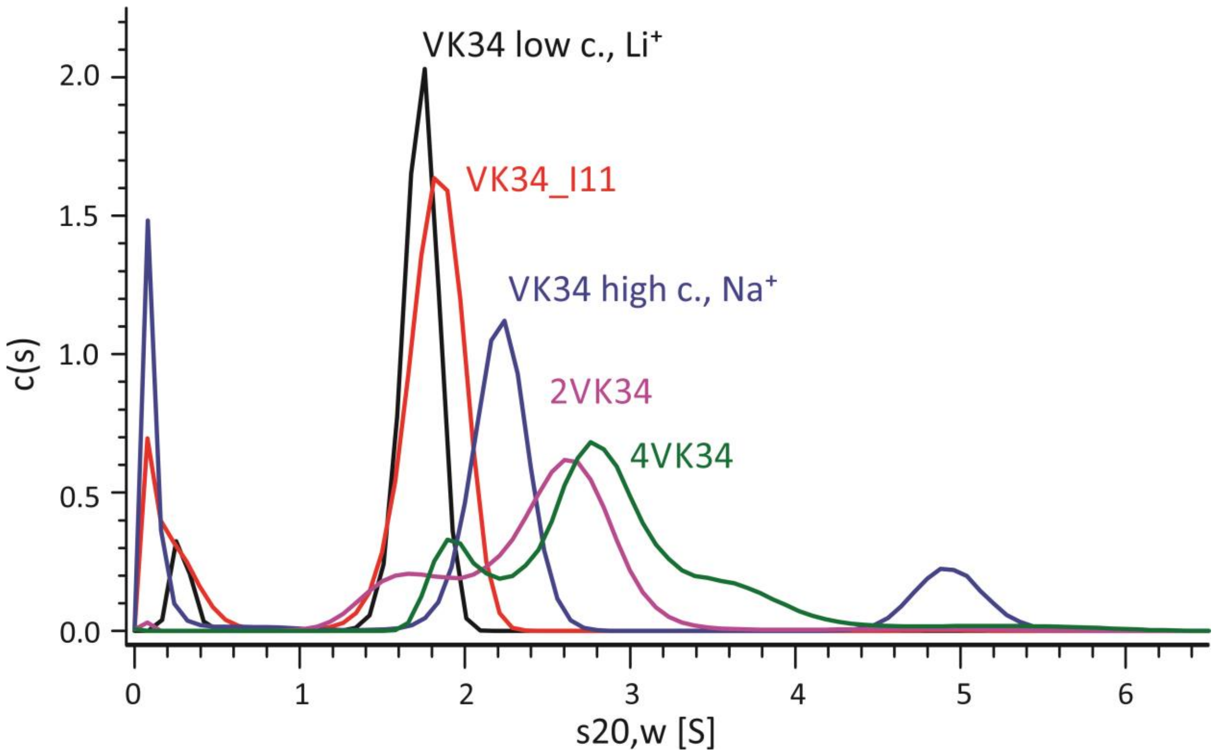

Supplementary Figure 3: The relative amount of the total absorbance c(s) of VK34, 2VK34, 4VK34 and VK34_I11 oligonucleotides as a function of sedimentation coefficient (s20,w[S]) corrected to density and viscosity of water at 20 °C. VK34 low c., Li^+ sample at 0.7 mM oligonucleotide concentration per strand, 0.5 M LiCl (recorded at 305 nm) sedimented as a dimeric species (30 nt) corresponding to a molecular weight of 10 kDa. **VK34_I11** sample at 2.1 µM oligonucleotide concentration per strand, 0.1 M LiCl (recorded at 260 nm) sedimented as a dimeric species (30 nt) corresponding to a molecular weight of 10 kDa. **VK34 high c., Na^+** sample at 1.5 mM oligonucleotide concentration per strand, 0.2 M NaCl (recorded at 310 nm) clearly sedimented as a much larger species than VK34 dimer, but a bit smaller than dimeric and monomeric 2VK34 and 4VK34 samples. Additionally, signal detected at around 5S probably corresponds to multimeric folds. **2VK34** sample at 1.6 µM oligonucleotide concentration per strand, 0.1 M LiCl (recorded at 260 nm) sedimented mostly as a dimeric species (62 nt) corresponding to a molecular weight of 19 kDa. **4VK34** sample at 0.5 µM oligonucleotide concentration per strand, 0.1 M LiCl (recorded at 260 nm) sedimented mostly as a monomeric species (63 nt) corresponding to a molecular weight of 21 kDa. All experiments were conducted at 5 °C.

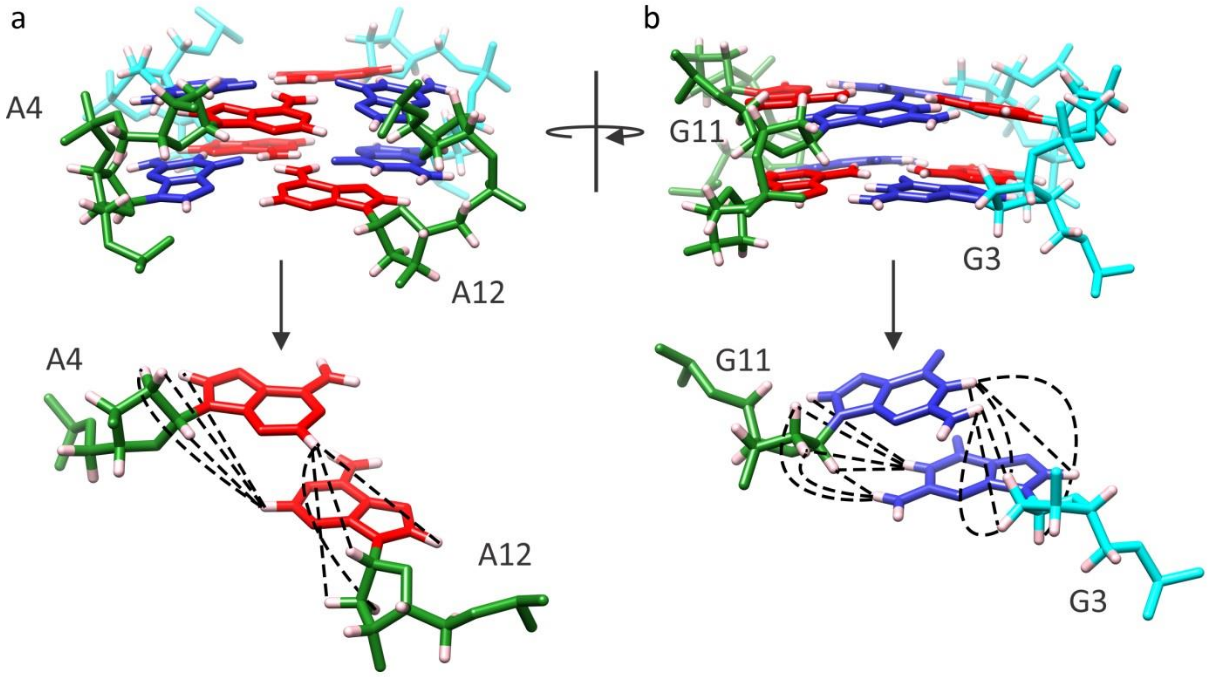

Supplementary Figure 4: Key NOE connectivities between residues that form two stacked GAGA-quartets in VK34 dimer. (a) Insight into the stacked pair of GAGA-quartets is shown on top, with a 90° rotated view shown in panel **(b)**. **(a)** NOE connectivities shown as dashed lines between protons of stacked A4 and A12 residues. **(b)** NOE connectivities between protons of stacked G3 and G11 residues. Location of the two stacked pairs of guanines as found in high-resolution structure VK34 dimer.

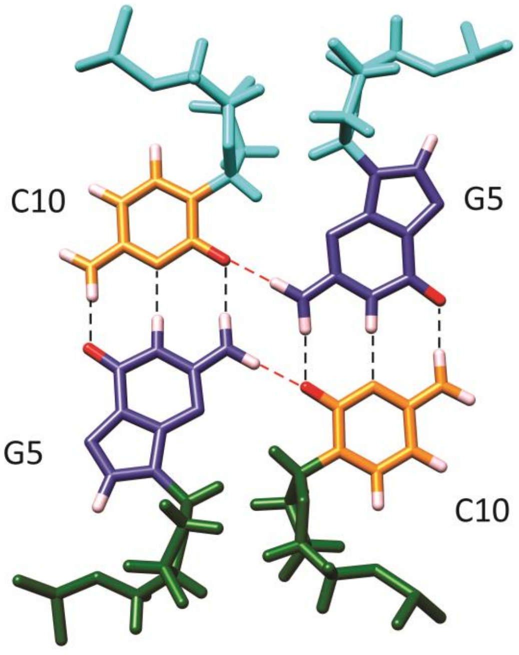

Supplementary Figure 5: Minor groove GCGC-quartet in VK34 dimer. Black dashed lines represent hydrogen bonds between guanine and cytosine residues in Watson-Crick geometry. Red dashed lines show hydrogen bonds connecting two G-C base pairs in a GCGC-quartet.

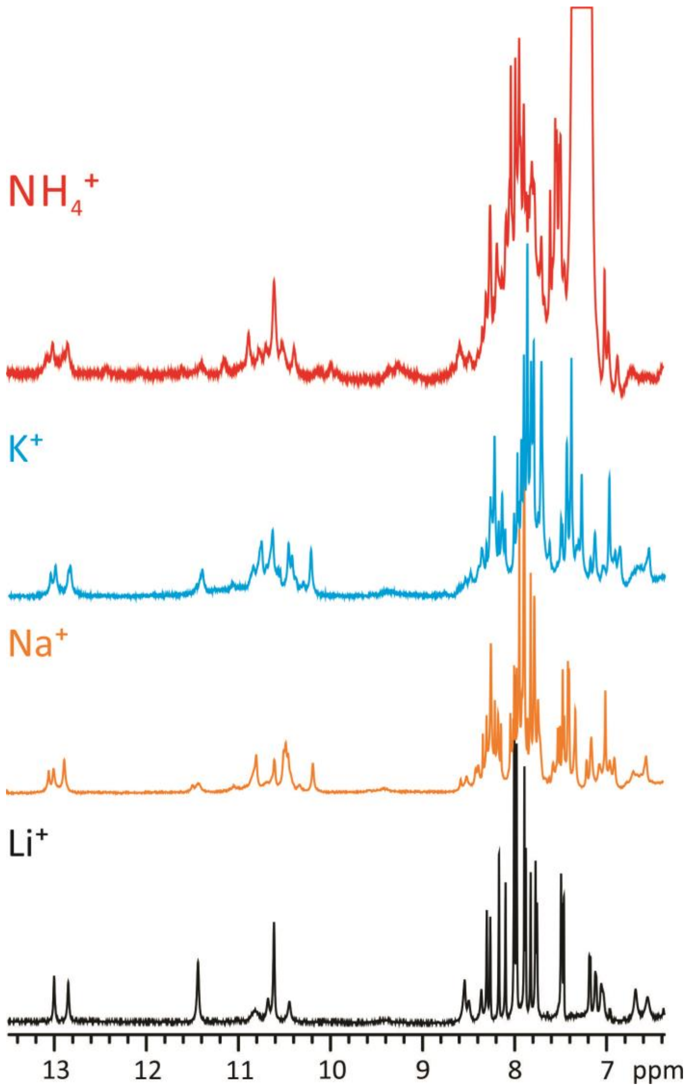

Supplementary Figure 6: ^{1}H NMR spectra of VK34 in the presence of different cations. The spectra were recorded at 1.0 mM oligonucleotide concentration per strand, pH 6.0, 0 °C and 100 mM cation concentrations on an 800 MHz spectrometer.

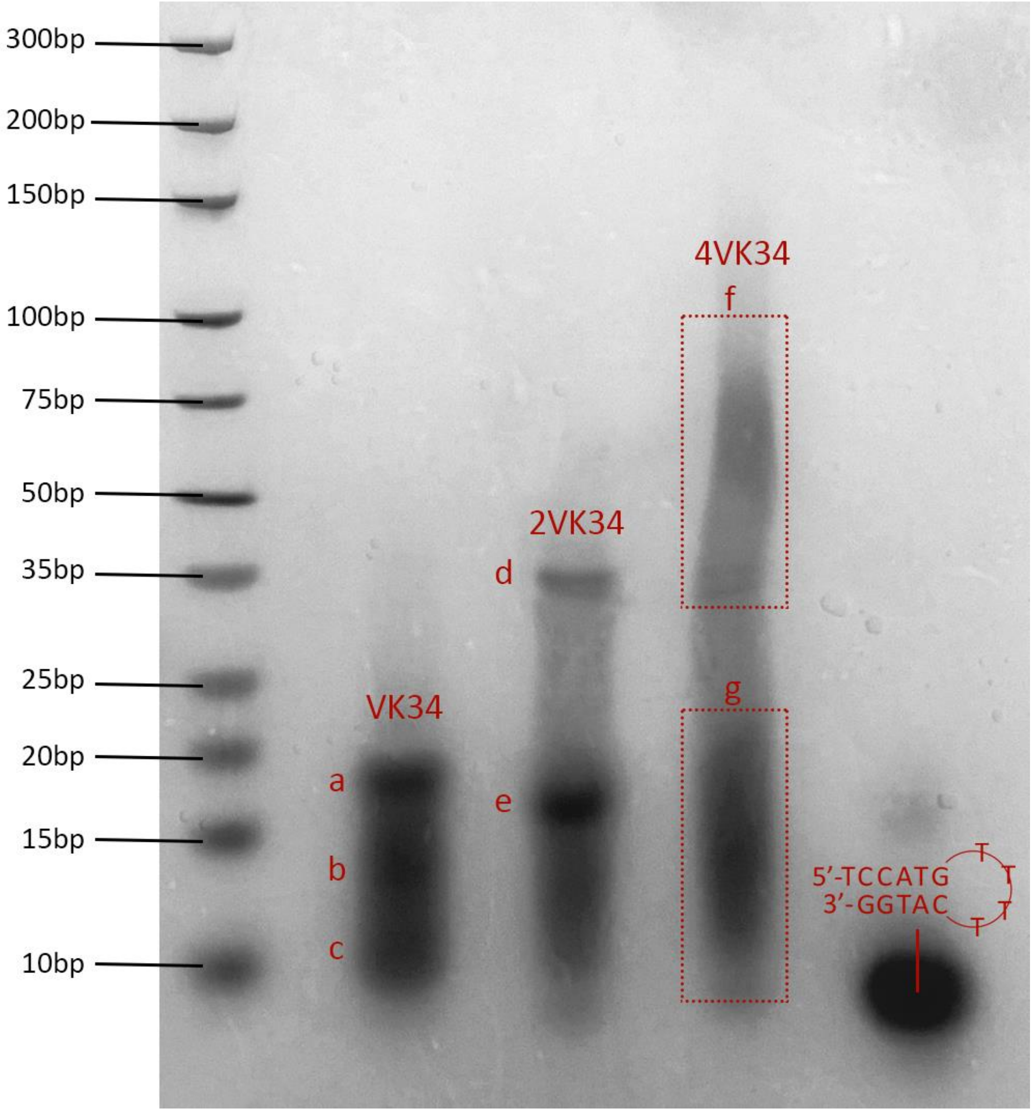

Supplementary Figure 7: Detection of VK34 tetramer, 2VK34 dimer and 4VK34 monomer by 10% native PAGE gel with 200 mM NaCl. Bands corresponding to DNA ladder with 300−10 base pairs are indicated on the left. **(a)** Band that corresponds to VK34 tetramer. **(b)** VK34 dimer. **(c)** Pre-folded duplex of VK34. **(d)** Trimer of 2VK34. **(e)** Dimer of 2VK34. **(f)** Bands attributed to dimer and higher associations of 4VK34. **(g)** Bands attributed to monomeric folds of 4VK34. Band that belongs to a control hairpin is indicated on the most right lane.

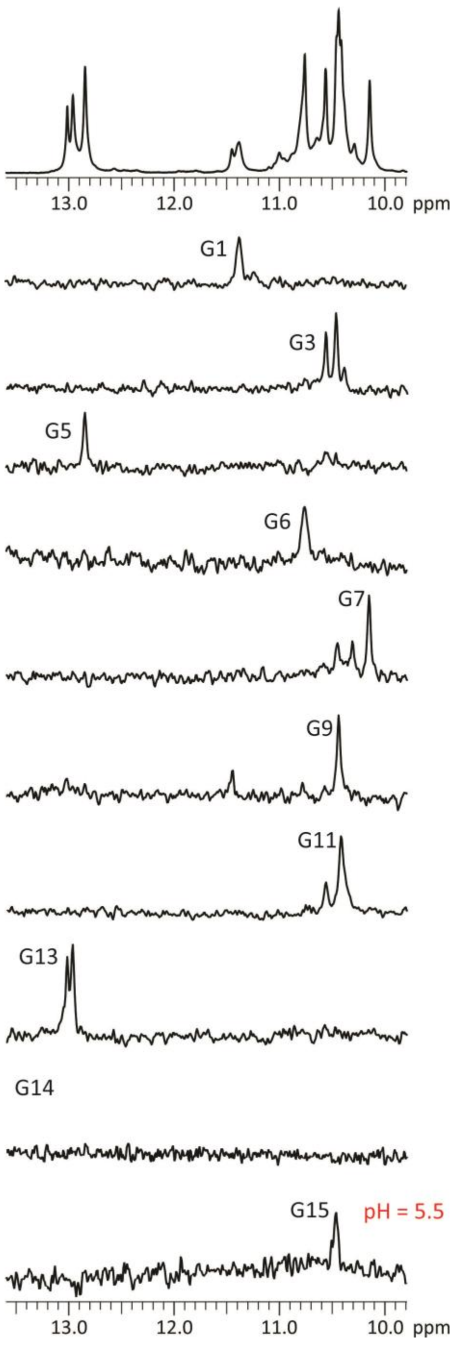

Supplementary Figure 8: Imino regions of 1D ^{1}H and 1D ^{15}N-edited HSQC spectra of partially (10%) residue-specific ^{15}N-labelled VK34 in the presence of 100 mM NaCl. Assignment of imino proton resonances is indicated next to the signals. The spectra were recorded at oligonucleotide concentrations ranging from 1.0 to 1.3 mM per strand, pH 6.0 and 0 °C on an 800 MHz and 600 MHz spectrometers. We were able to observe G15 imino proton resonance at pH 5.5. No G14 imino proton resonance was observed. We observe some apparent doublets and multiplets for some imino protons due to slight breaking of symmetry between top and bottom parts of VK34 tetramer.

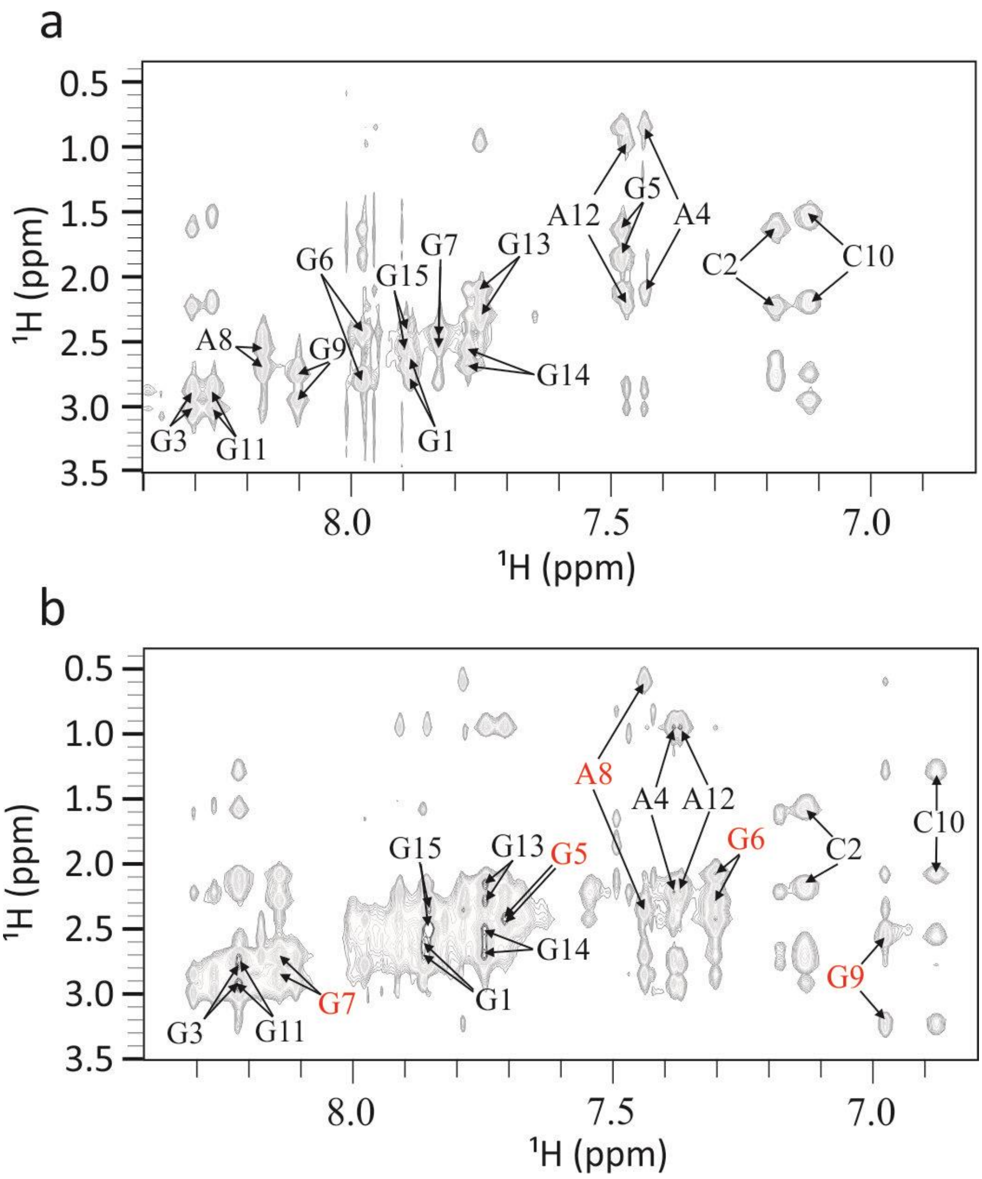

Supplementary Figure 9: Comparison of H2'/H2"-aromatic regions of NOESY spectra of VK34 dimer and tetramer. **(a)** H2'/H2"-aromatic region of NOESY spectrum (mixing time of 200 ms) of VK34 dimer recorded at 0.4 mM oligonucleotide concentration, pH 6.0 and 100 mM LiCl. **(b)** H2'/H2"-aromatic region of NOESY spectrum (mixing time of 150 ms) of VK34 tetramer recorded at 1.0 mM oligonucleotide concentration, pH 6.0 and 100 mM NaCl. Residues in NOESY spectrum of the tetrameric fold that have similar NOE fingerprints with residues in the dimeric fold are labelled in black. Residues with NOE fingerprints unique to the tetrameric fold are shown in red.

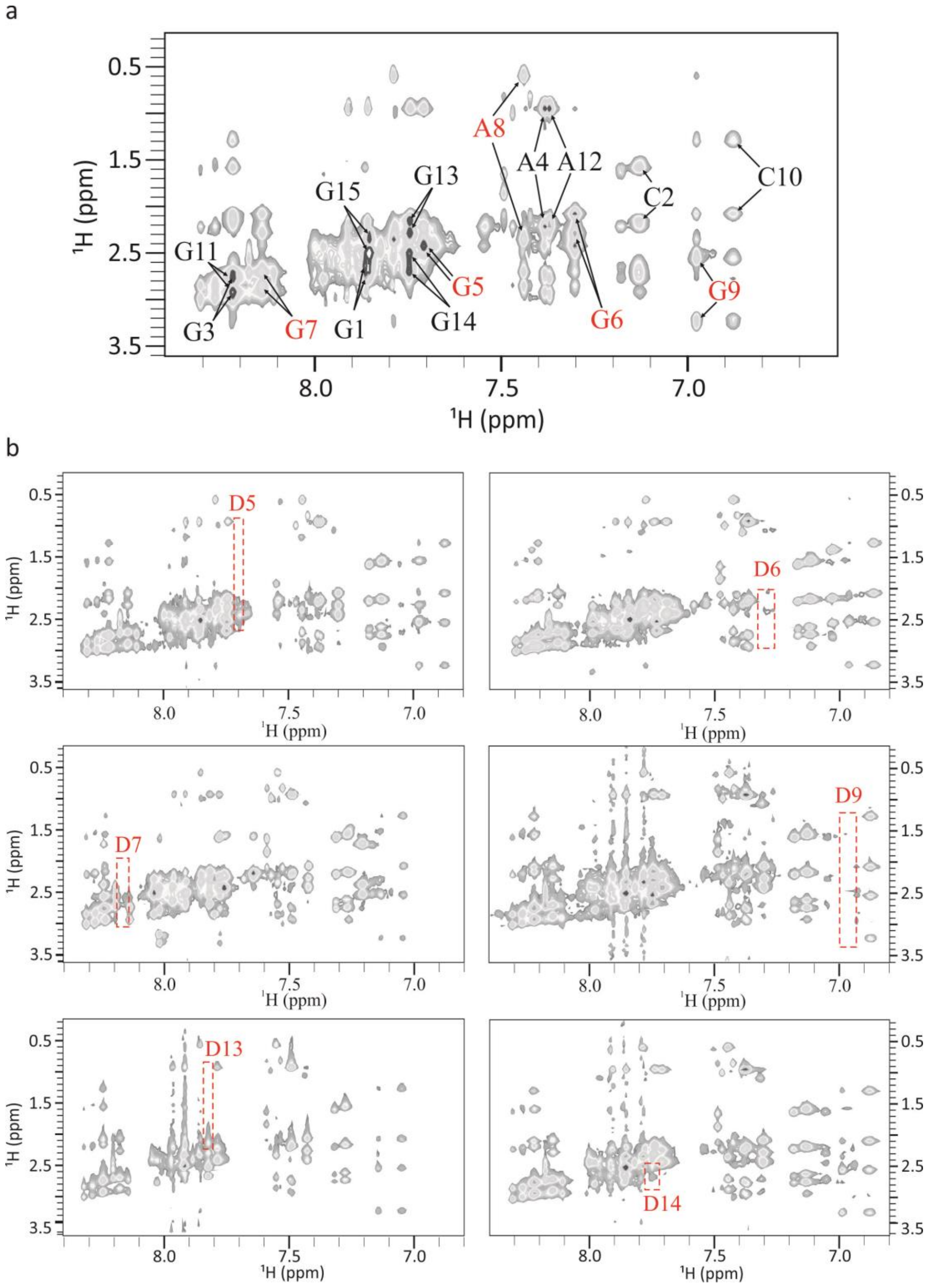

Supplementary Figure 10: Assignment of G5, G6, G7, G9, G13 and G14 aromatic proton resonances of D8-deuterated VK34 tetramer in the presence of Na^{+} ions. (a) H2'/H2"-aromatic region of NOESY spectrum (mixing time of 150 ms) recorded at 1.0 mM oligonucleotide concentration, pH 6.0 and 100 mM NaCl. Residues with NOE fingerprints unique to the tetrameric fold are shown in red. **(b)** H2'/H2"-aromatic regions of NOESY spectra (mixing time of 150 ms) of completely (100%) D8 residue-specific labelled oligonucleotides at pH 6.0 and 100 mM NaCl. Red dashed squares show regions with the missing NOE cross-peaks. Individual spectra were acquired on residue specifically D8-labeled guanine VK34 marked with the letter D followed by a position in the sequence.

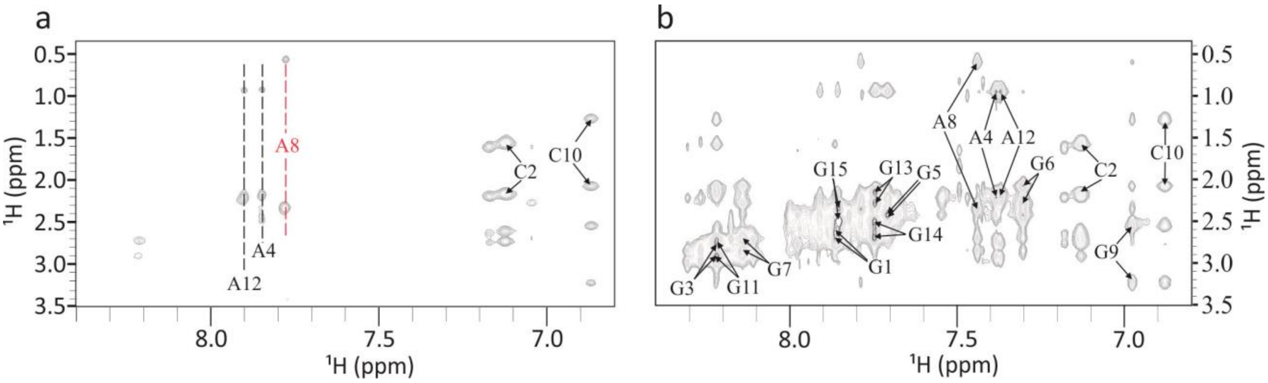

Supplementary Figure 11: Assignment of adenine H2 and cytosine H6 resonances of completely D8-deuterated VK34 tetramer. (a) H2'/H2"-aromatic region of NOESY spectrum (mixing time of 150 ms) recorded on VK34 with all G and A completely (100%) D8 labelled at 1.0 mM oligonucleotide concentration, pH 6.0 and 100 mM NaCl. **(b)** H2'/H2"-aromatic region of NOESY spectrum (mixing time of 150 ms) recorded at 1.0 mM oligonucleotide concentration, pH 6.0 and 100 mM NaCl. The black dashed lines connect the cross-peaks between H2 and sugar proton resonances. The red dashed line connects cross-peaks between H2 and sugar proton resonances which are unique to the tetrameric VK34 fold.

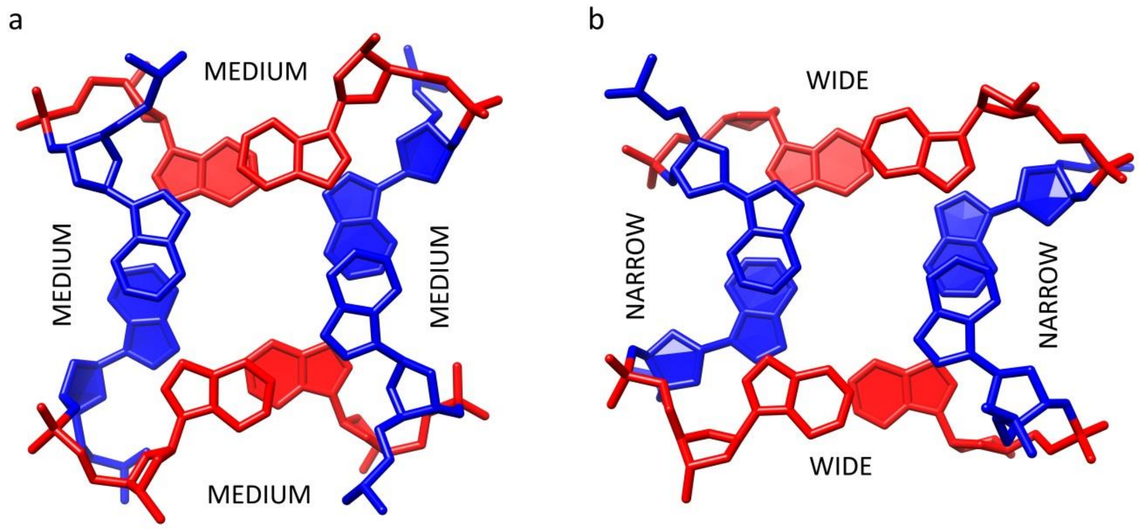

Supplementary Figure 12: Stacked GAGA-quartets with different grooves. (a) Top view of two stacked GAGA-quartets with four medium grooves found in VK34 dimer. **(b)** Top view of two stacked GAGA-quartets with alternating narrow and wide grooves found in VK34 tetramer.

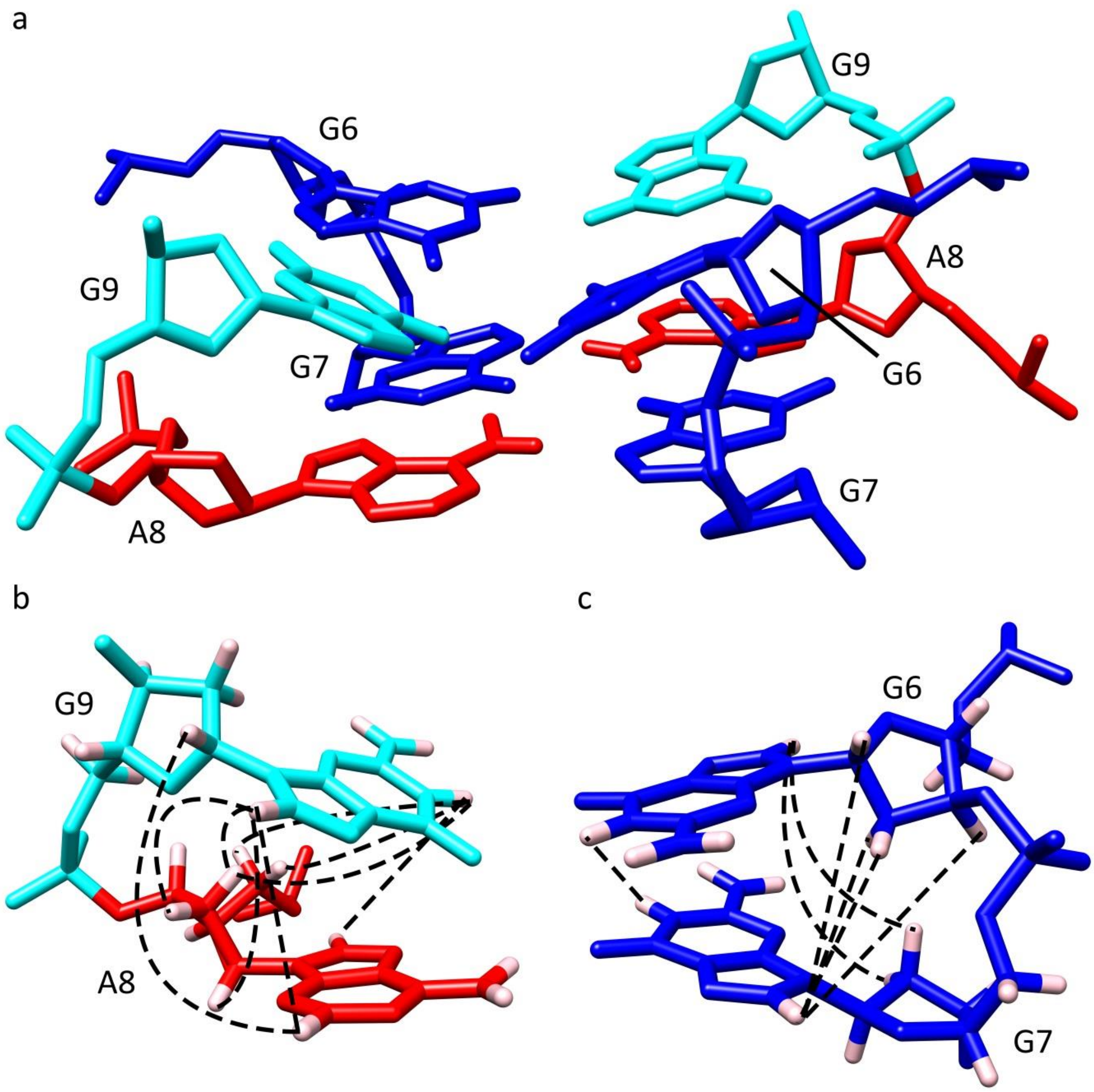

Supplementary Figure 13: G6-G9-G6-G9 quartet stacked on G7-A8-G7-A8 quartet inside VK34 tetramer. (a) G6-G9-G6-G9 quartet. **(b)** Observed NOE contacts between A8 and G9 residues. **(c)** Observed NOE contacts between G6 and G7 residues.

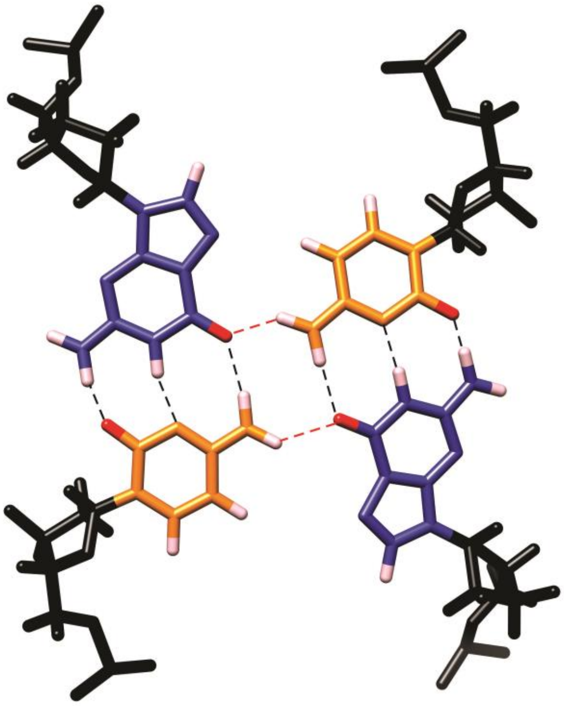

Supplementary Figure 14: Major groove GCGC-quartet in VK34 tetramer. Black dashed lines represent hydrogen bonds between guanine and cytosine residues in Watson-Crick geometry. Red dashed lines show which hydrogen bonds that connect two G-C base pairs in a major groove GCGC-quartet. The guanine bases are colored blue and cytosine, orange. Hydrogen and O6 atoms are colored white and red, respectively.

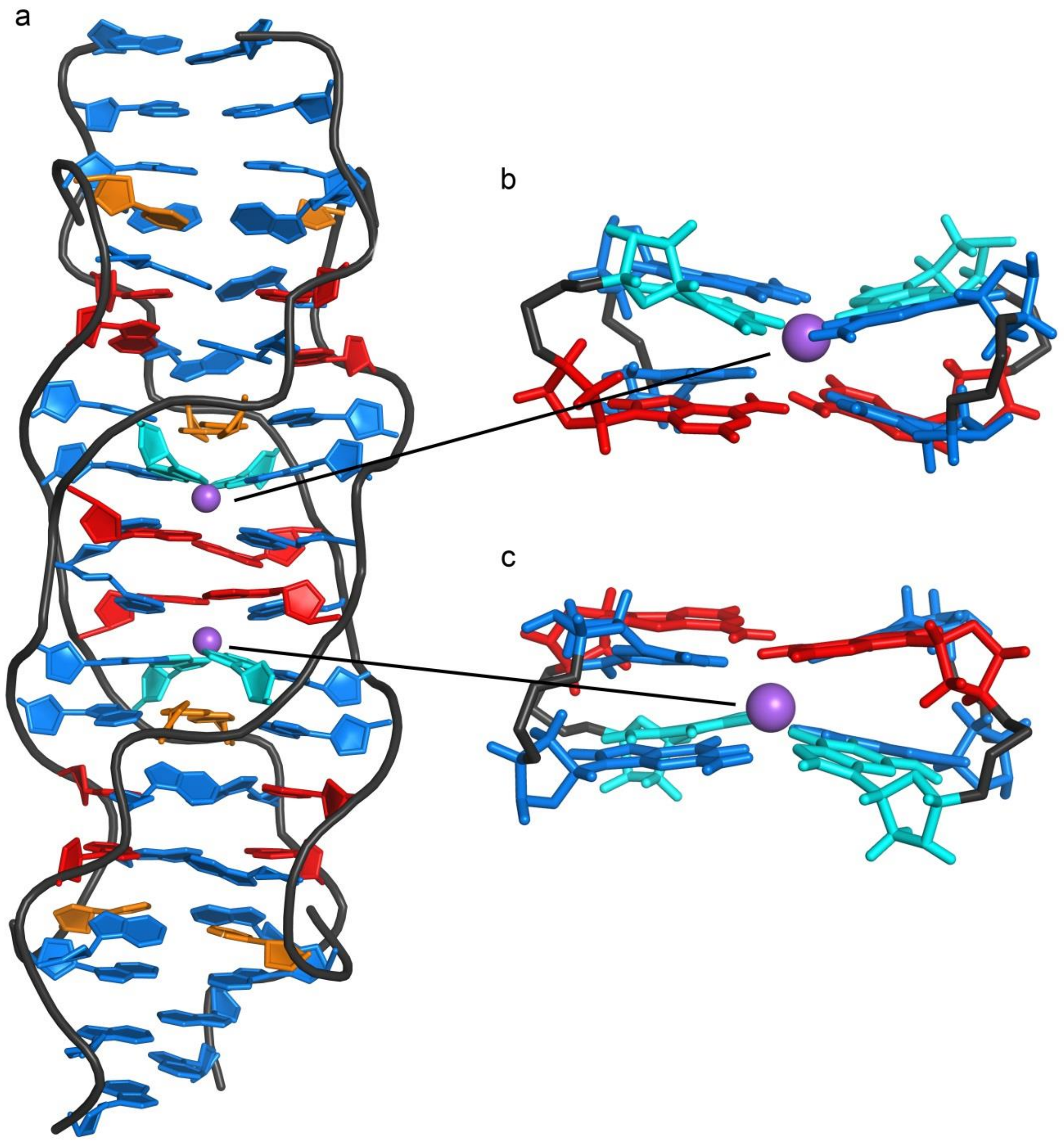

Supplementary Figure 15: Structure of VK34 tetramer refined with explicit water model, randomly distributed cations and without planarity restraints with two localized Na^+ cations. (a) Depiction of the whole VK34 structure with two localized Na^+ cations. **(b)** Na^+ cation localized between the top G6-G9-G6-G9 and G7-A8-G7-A8 quartets. **(c)** Na^+ cation localized between the bottom G6-G9-G6-G9 and G7-A8-G7-A8 quartets.

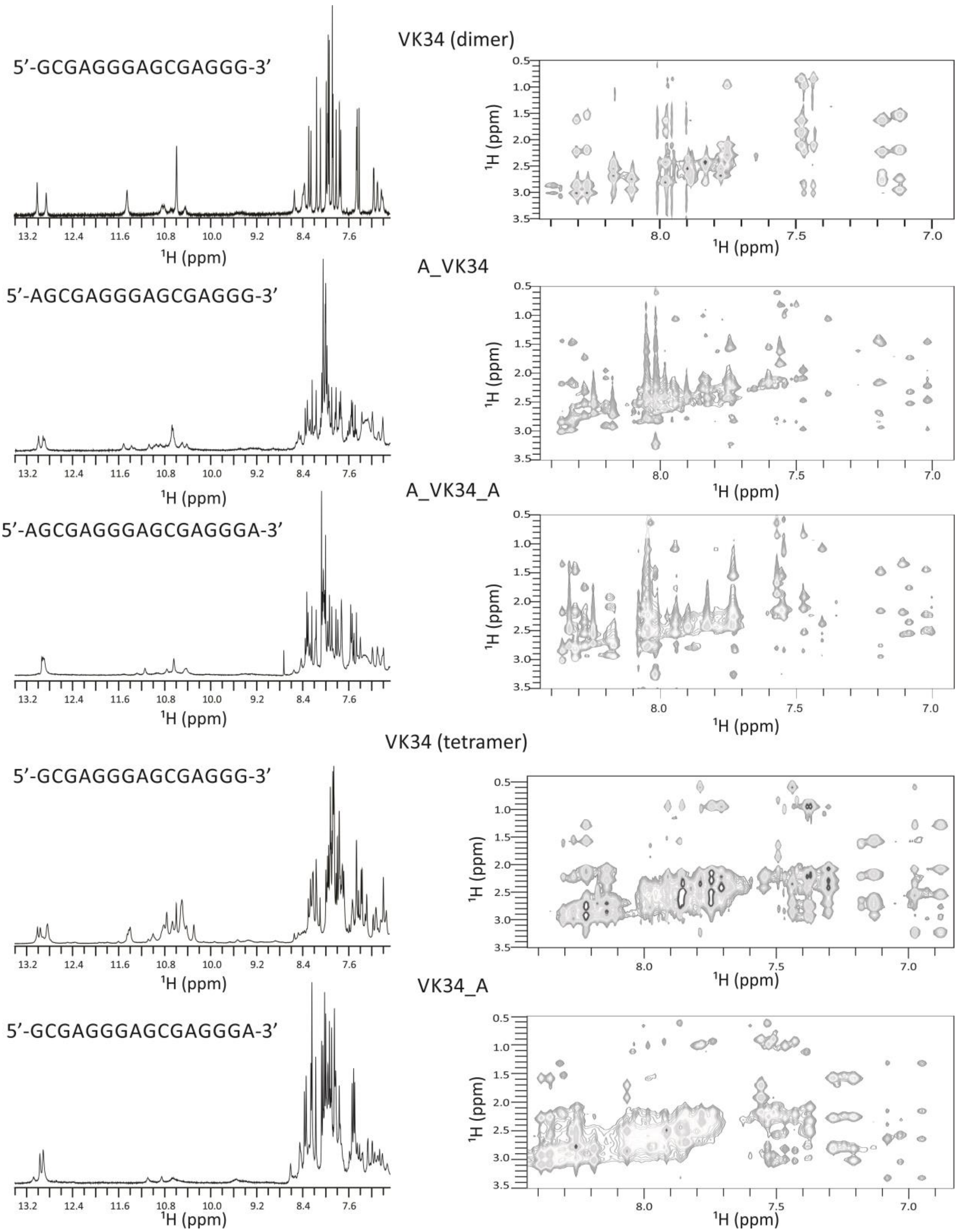

Supplementary Figure 16: Imino and aromatic regions of 1D ^{1}H and H2’/H2”-aromatic regions of 2D NOESY spectra of A_VK34, A_VK34_A and VK34_A folds as well as VK34 dimeric and tetrameric folds. Sequences and 1D ^{1}H spectra of A_AVK34, A_VK34_A and VK34_A oligonucleotides are indicated in red, green and blue, respectively. The corresponding NOESY spectra are shown on the right side of the figure.

a

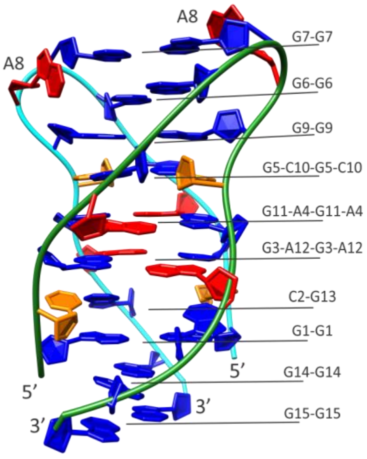

b

Step	Rise (Å)	Twist (deg)
G7-G7/<br>G6-G6	3.8 ± 0.3	24±2
G6-G6/<br>G9-G9	3.5 ± 0.2	51±3
G9-G9/<br>G5-C10-G5-C10	3.4 ± 0.3	51±1
G5-C10-G5-C10/<br>G11-A4-G11-A4	3.5 ± 0.3	5±1
G11-A4-G11-A4/<br>G3-A12-G3-A12	3.6 ± 0.2	25±1
G3-A12-G3-A12/<br>C2-G13,C2-G13 plane	3.7 ± 0.3	4±2
C2-G13,C2-G13 plane/<br>G1-G1	3.6 ± 0.2	54±4
G1-G1/<br>G14-G14	3.6 ± 0.3	60±5
G14-G14/<br>G15-G15	3.5 ± 0.1	23±3

c

Base pairs	Buckle (deg)	Propeller twist (deg)
G7-G7	~0	~0
G6-G6	~0	~0
G9-G9	~0	~0
G5-C10, G5-C10	10±2	~0
G11-A4, G11-A4	8±2	~0
G3-A12, G3-A12	~0	~0
C2-G13, C2-G13	28±2	14±4
G1-G1	~0	~0
G14-G14	~0	~0
G15-G15	*7±4	~0

Observed in four out of ten structures*

d

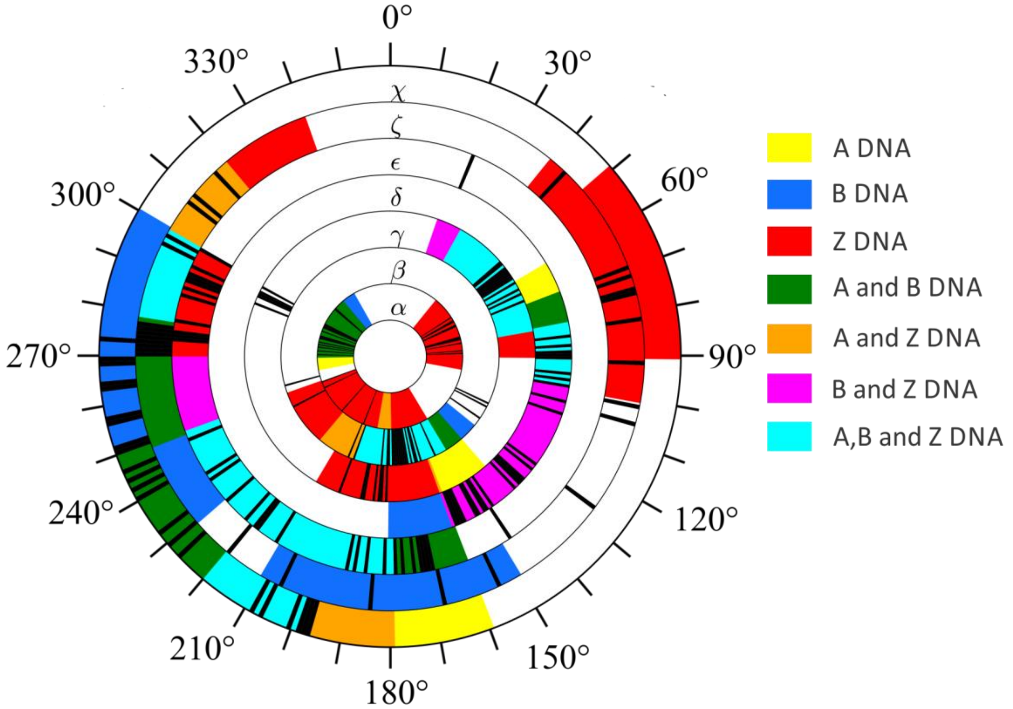

Supplementary Figure 17: Base step, base-pair and torsion angle analysis in a family of ten structures of VK34 dimer. (a) Structure of VK34 dimer with indicated base pairs and planes defined by GAGA-quartets, GCGC-quartets and G-C base pairs. **(b)** Rise and twist between base pairs and planes. **(c)** Table of base pair buckle and propeller twist. **(d)** Conformational wheel for α, β, γ, δ, ε, ζ and χ torsion angles for all residues.

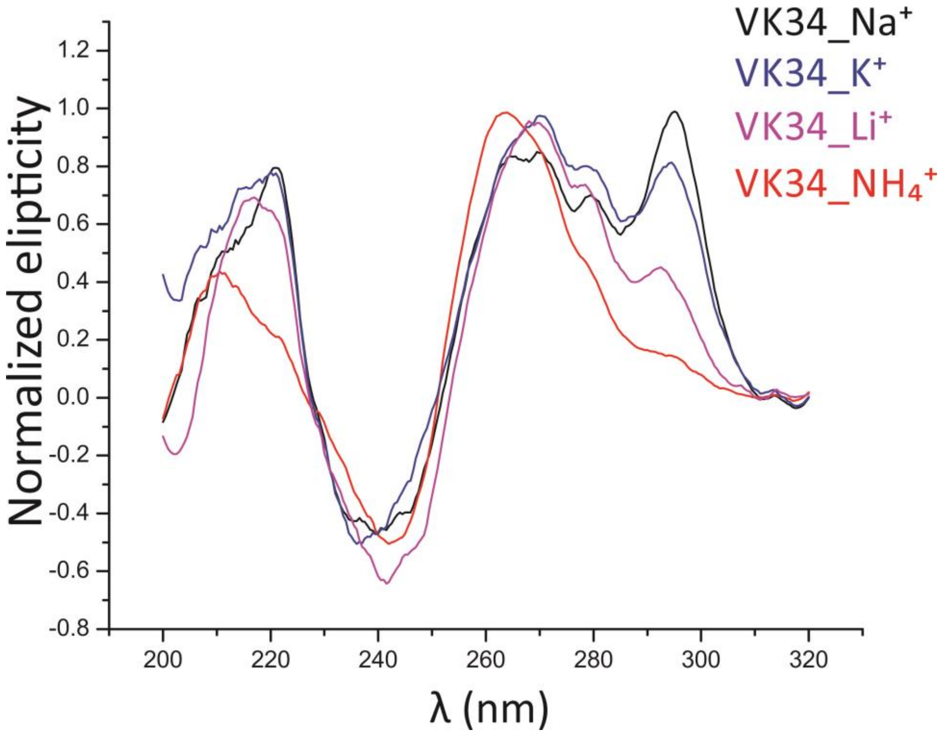

Supplementary Figure 18: CD spectra of VK34 in the presence of different cations. All CD spectra were recorded at concentrations close to 1.0 mM oligonucleotide concentrations, 100 mM salt solutions (NaCl, KCl, LiCl and NH_4Cl) in a 0.01 cm path-length cell at pH 6.0 and 0 °C.

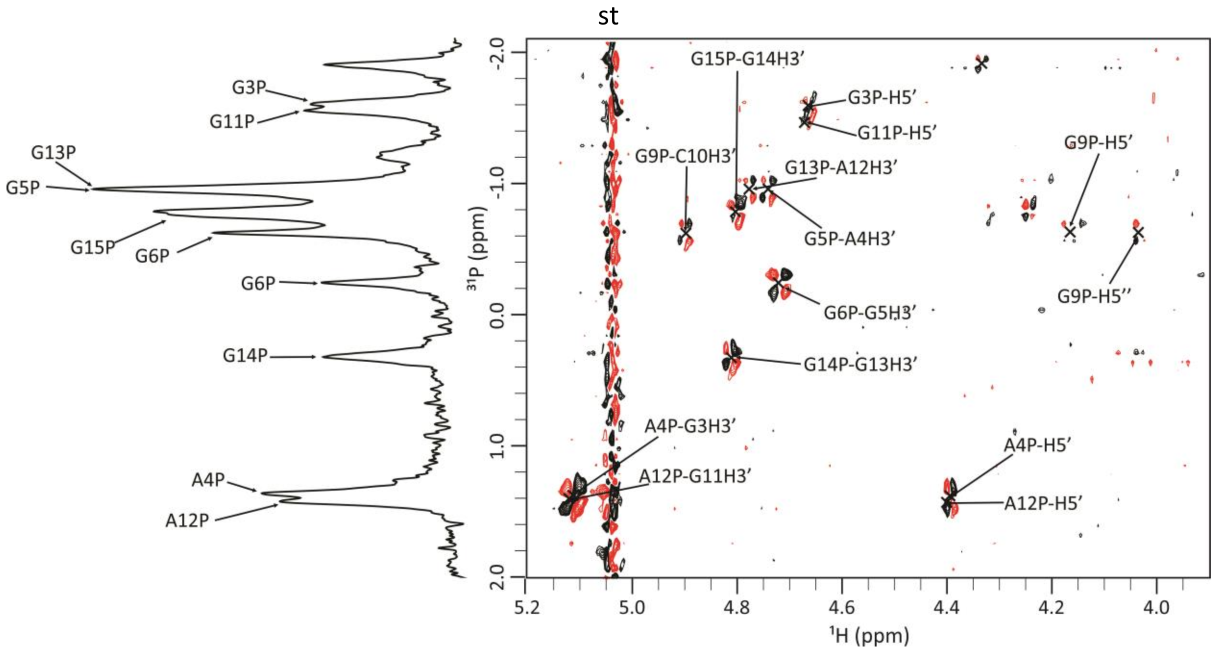

Supplementary Figure 19: 1D ^{31}P NMR spectrum of the VK34 dimer shown alongside a 2D ^{1}H–^{31}P COSY spectrum. Assignment of individual cross-peaks is indicated. Both spectra were recorded at 0.8 mM oligonucleotide concentration per strand, pH 6 and 0 °C on a 600 MHz spectrometer.

a

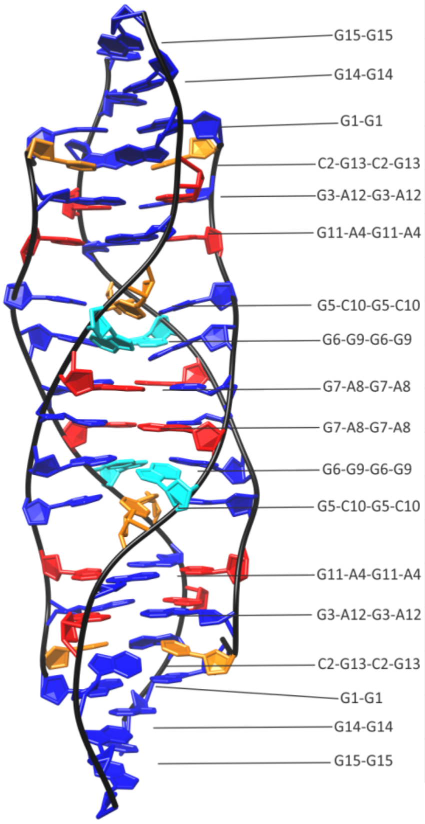

b

Step	Rise (Å)	Twist (deg)
G15-G15/ G14-G14	3.8 ± 0.3	11 ± 4
G14-G14/ G1-G1	3.4 ± 0.1	40 ± 4
G1-G1/ C2-G13-C2-G13	3.2 ± 0.2	43 ± 1
C2-G13-C2-G13/ G3-A12-G3-A12	3.6 ± 0.2	10 ± 1
G3-A12-G3-A12/ G11-A4-G11-A4	3.6 ± 0.2	22 ± 1
G11-A4-G11-A4/ G5-C10-G5-C10	5.0 ± 0.4	19 ± 3
G5-C10-G5-C10/ G6-G9-G6-G9	3.1 ± 0.1	21 ± 2
G6-G9-G6-G9/ G7-A8-G7-A8	4.2 ± 0.4	17 ± 4
G7-A8-G7-A8/ G7-A8-G7-A8	3.5 ± 0.1	27 ± 1
G7-A8-G7-A8/ G6-G9-G6-G9	4.2 ± 0.2	22 ± 2
G6-G9-G6-G9/ G5-C10-G5-C10	3.1 ± 0.3	21 ± 1
G5-C10-G5-C10/ G11-A4-G11-A4	4.8 ± 0.3	21 ± 1
G11-A4-G11-A4/ G3-A12-G3-A12	3.6 ± 0.3	22 ± 1
G3-A12-G3-A12/ C2-G13-C2-G13	3.7 ± 0.5	14 ± 2
C2-G13-C2-G13/ G1-G1	2.9 ± 0.1	44 ± 2
G1-G1/ G14-G14	3.5 ± 0.2	44 ± 2
G14-G14/ G15-G15	3.8 ± 0.3	17 ± 3

c

Base pairs	Buckle (deg)	Propeller twist (deg)	Quartet buckle (deg)
G15-G15	28 ± 2	12 ± 3	N.A.
G14-G14	19 ± 1	23 ± 3	N.A.
G1-G1	16 ± 2	9 ± 3	N.A.
C2-G13-C2-G13	8 ± 1	~0	29 ± 2
G3-A12-G3-A12	~0	~0	~0
G11-A4-G11-A4	~0	~0	~0
G5-C10-G5-C10	~0	11 ± 3	10 ± 6
G6-G9-G6-G9	~0	~0	14 ± 4
G7-A8-G7-A8	~0	~0	~0
G7-A8-G7-A8	~0	~0	~0
G6-G9-G6-G9	~0	~0	14 ± 5
G5-C10-G5-C10	~0	11 ± 3	11 ± 3
G11-A4-G11-A4	~0	~0	12 ± 2*
G3-A12-G3-A12	9 ± 2	~0	18 ± 3**
C2-G13-C2-G13	8 ± 1	~0	27 ± 3
G1-G1	13 ± 2	7 ± 3	N.A.
G14-G14	18 ± 2	10 ± 5	N.A.
G15-G15	18 ± 1	12 ±6	N.A.

Observed in only half of the structures*

Observed in only 75% of the structures**

d

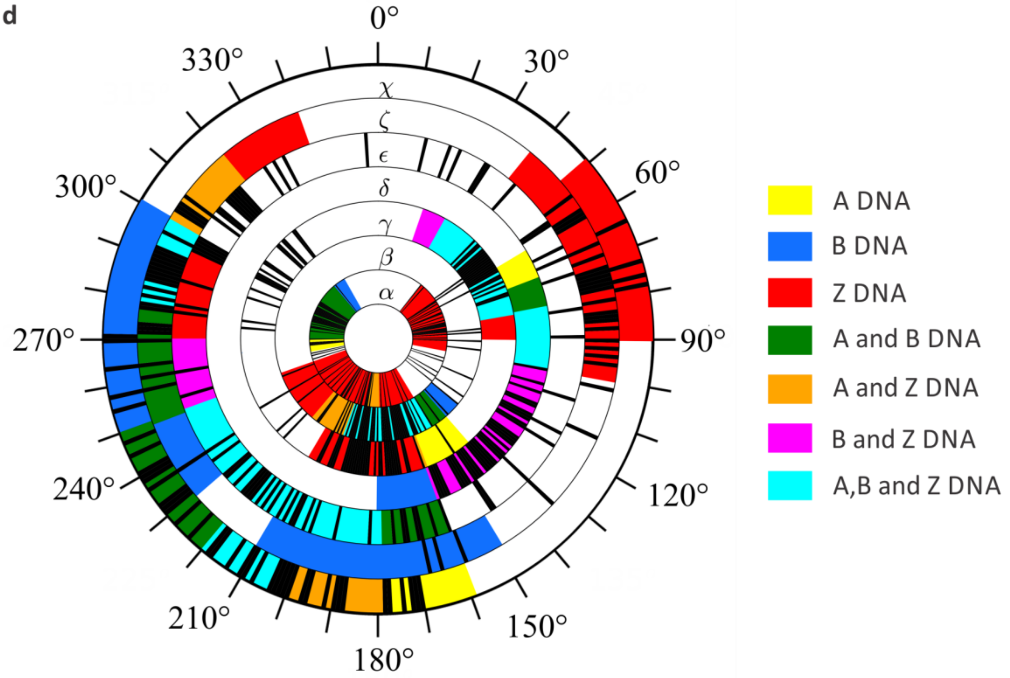

Supplementary Figure 20: Base step, base-pair and torsion angle analysis of a family of ten structures of VK34 tetramer. (a) Structure of VK34 tetramer with indicated base pairs and planes defined by GAGA-quartets and GCGC-quartets. **(b)** Table of rise and twist between base pairs and planes. **(c)** Table of base pair buckle, propeller twist and quartet buckle. **(d)** Conformational wheel for torsion angle values of α, β, γ, δ, ε, ζ and χ for all residues.

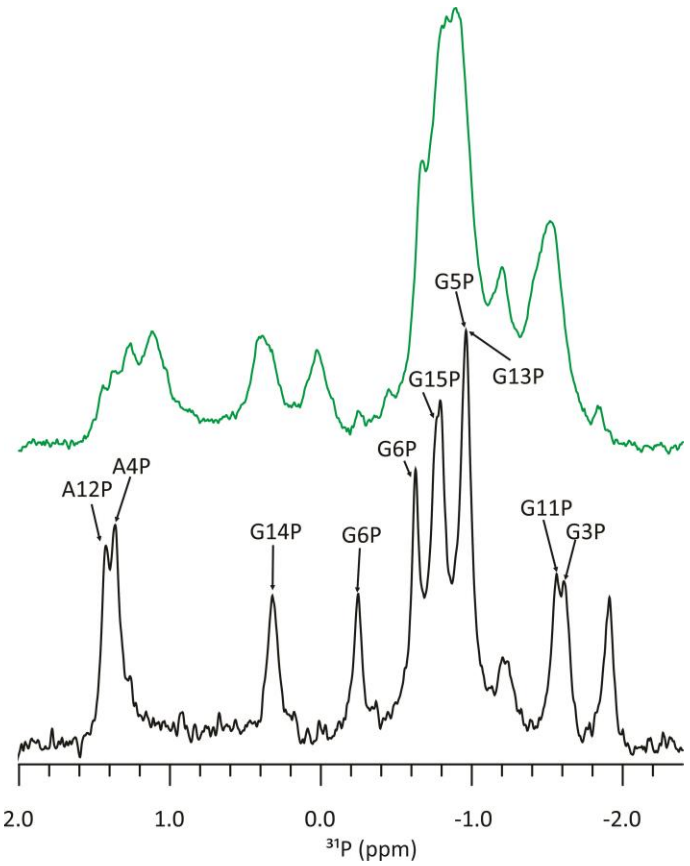

Supplementary Figure 21: 1D ^{31}P NMR spectra of the VK34 dimer (black) and VK34 tetramer (green). Spectrum of VK34 dimer was recorded at 0.4 mM oligonucleotide concentration per strand, 100 mM LiCl, pH 6.0 and spectrum of VK34 tetramer was recorded 1.2 mM oligonucleotide concentration per strand, 100 mM NaCl and pH 6.0. Both spectra were recorded at 0° C on a 600 MHz spectrometer. Assignment of individual signals is indicated.

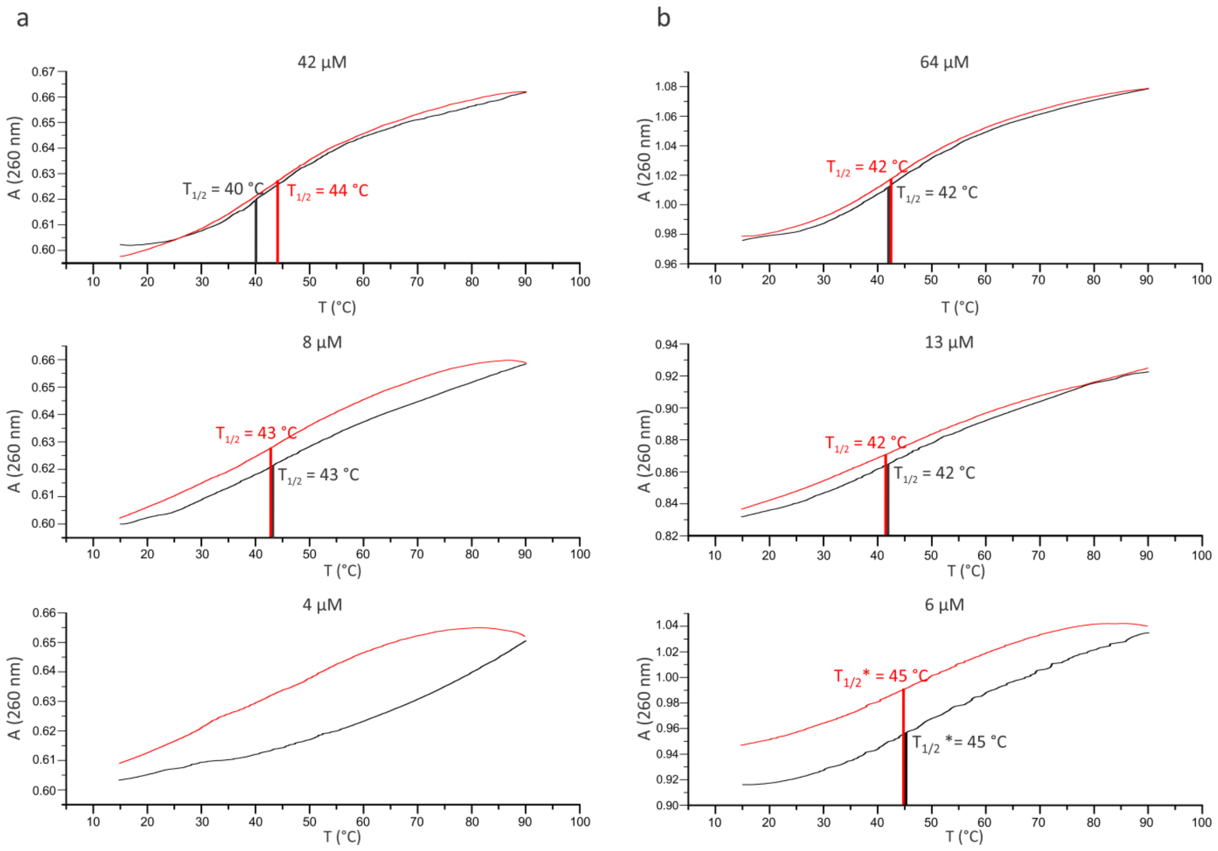

Supplementary Figure 22: Temperature-dependent UV experiments for (a) VK34 dimer and (b) VK34 tetramer. The spectra were recorded at 100 mM LiCL and 100 mM NaCl concentrations for the VK34 dimer and VK34 tetramer, respectively. The unfolding/refolding processes were followed between 15 and 90 °C by measuring absorbance at 260 nm with a scanning rate of 0.3 °C min^{-1}. The oligonucleotide concentrations are indicated on top of individual spectrum. The heating curve is labeled in black and the cooling curve in red. The temperatures of midtransitions are indicated with $T_{1/2}$ (black for heating, red for cooling). The asterisks indicate broad transitions.

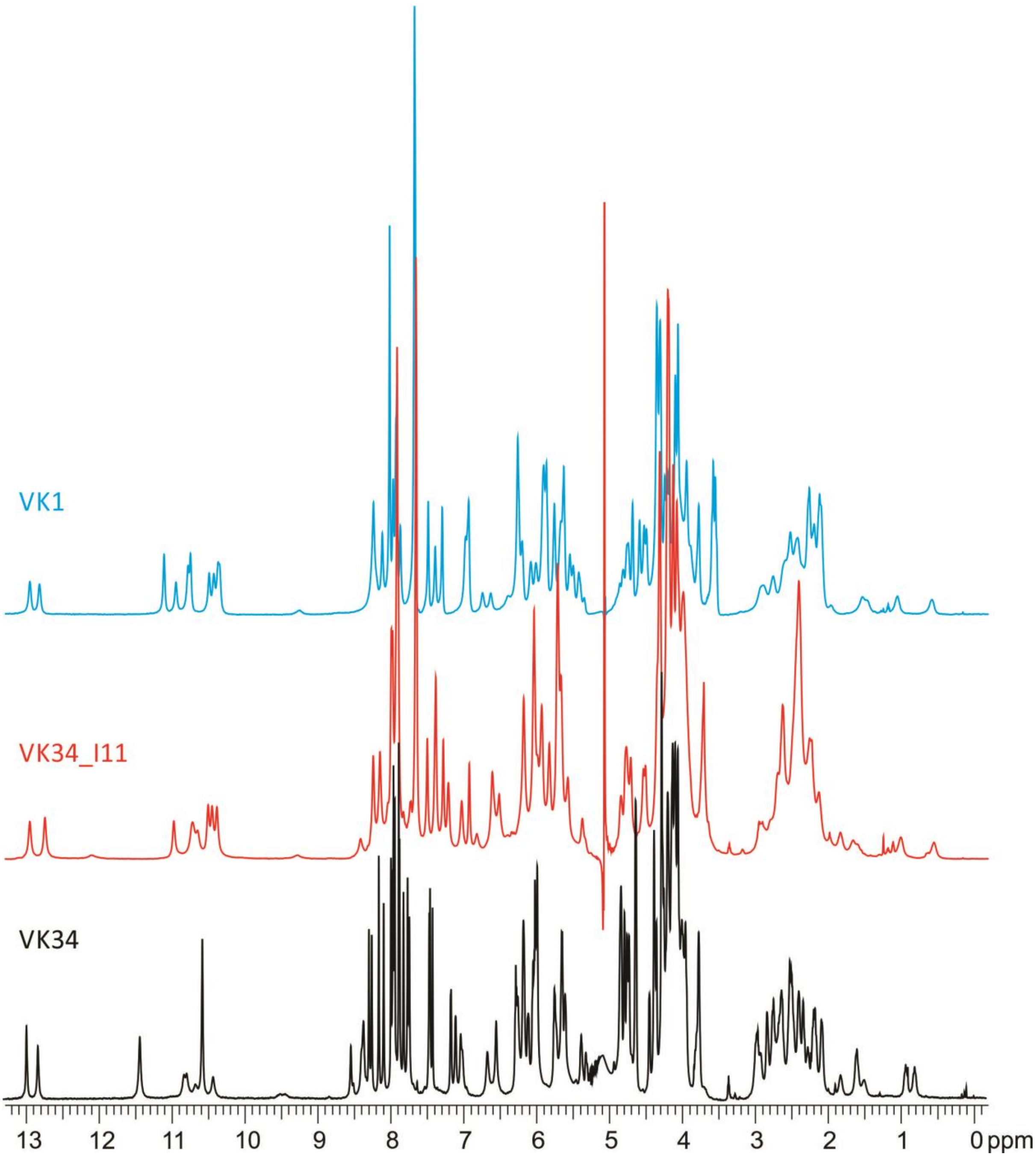

Supplementary Figure 23: ^{1}H NMR spectra of VK34, VK34_I11 and VK1. The spectra were recorded at 1.2 mM (VK1 and VK34_I11) and 0.8 mM (VK34) oligonucleotide concentration per strand, pH 6.0, 0 °C and 100 mM LiCl concentration on an 800 MHz spectrometer.

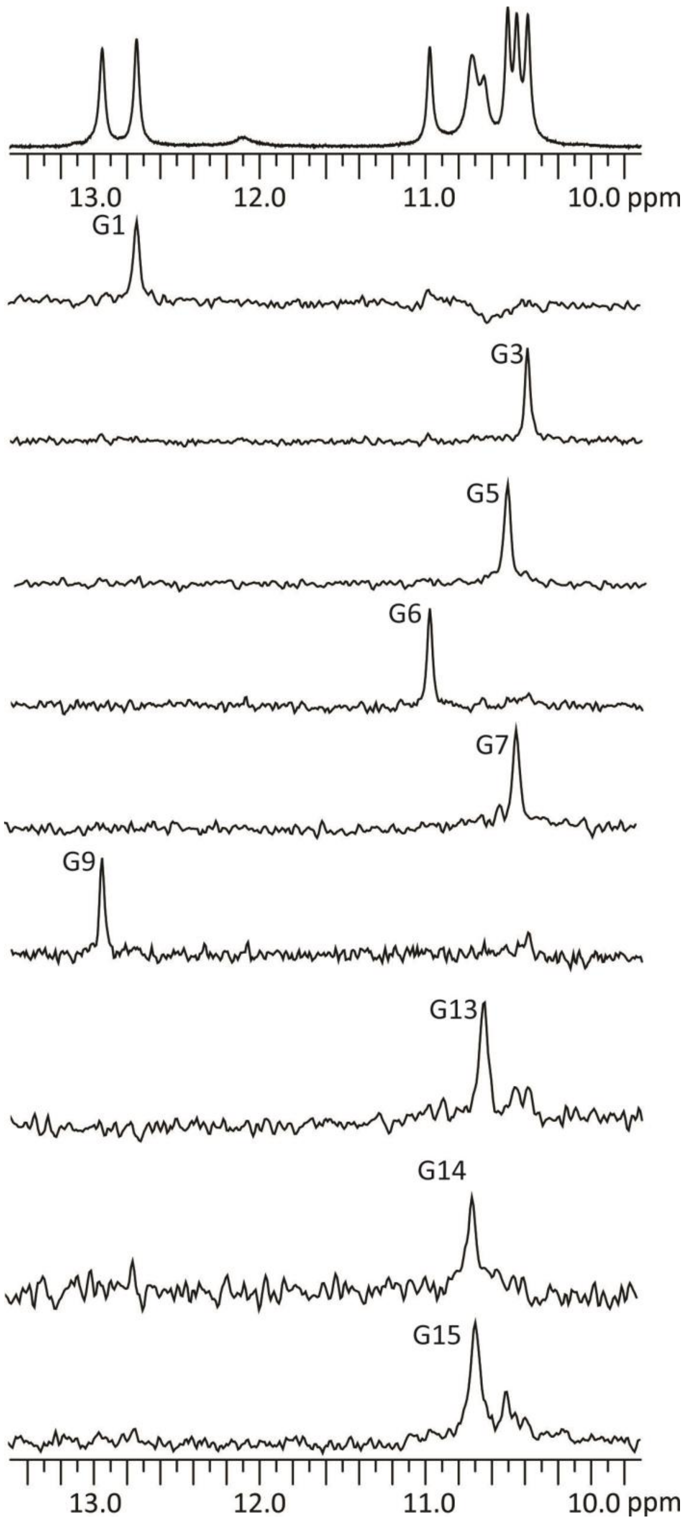

Supplementary Figure 24: Assignment of H1 proton resonances of VK34_I11. The imino region of 1D ^{1}H NMR spectrum is positioned above 1D ^{15}N-edited HSQC spectra acquired on partially (10%) residue-specific ^{15}N-labelled oligonucleotides. Assignment of imino proton resonances is indicated next to the signals. The spectra were recorded at oligonucleotide concentrations ranging from 0.8 to 1.2 mM per strand, pH 6.0, 0 °C and 100 mM LiCl concentration on an 800 MHz and 600 MHz spectrometers.

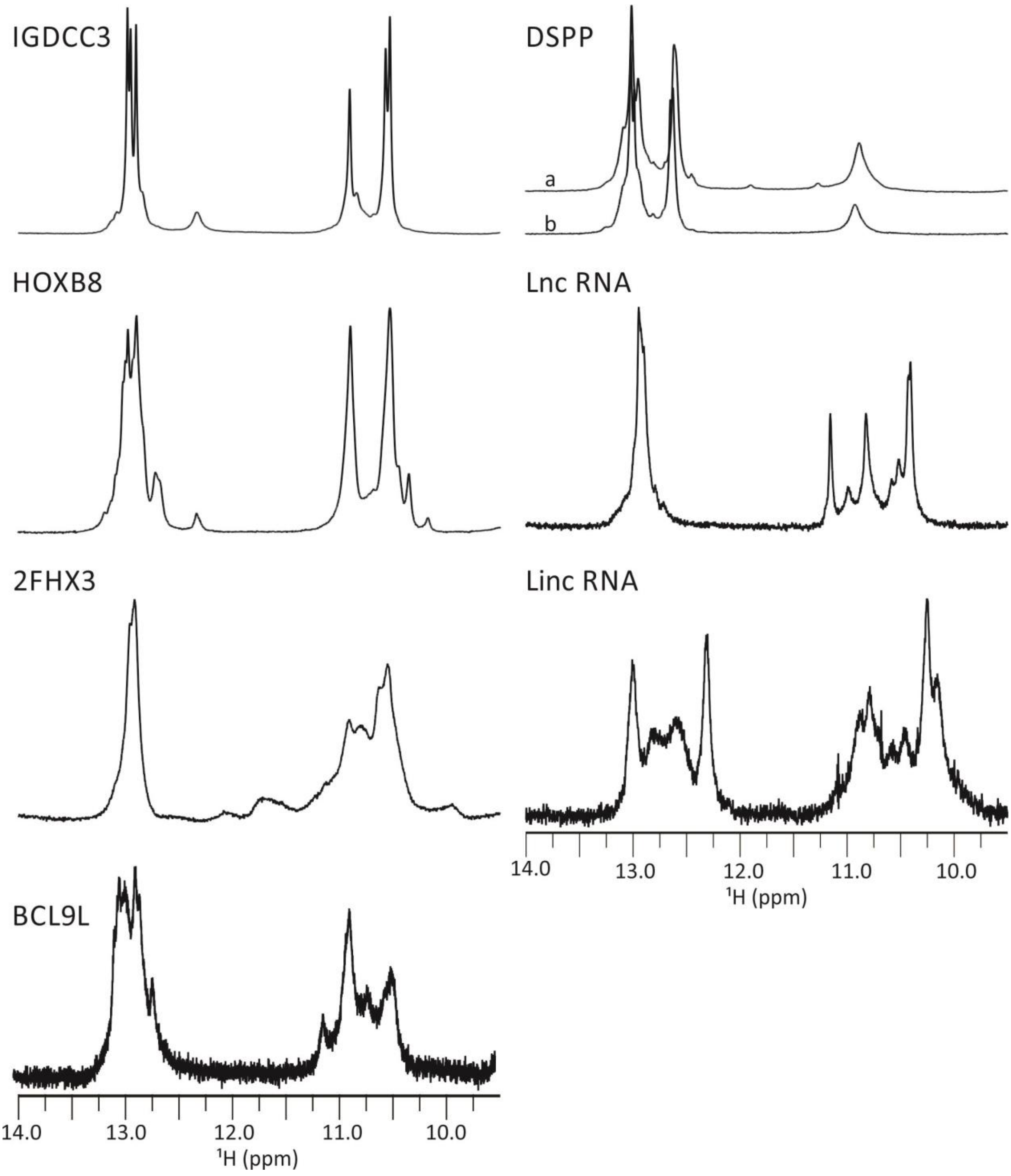

Supplementary Figure 25: The NMR spectra of oligonucleotides containing AGCGA repeats found in different human genes. 1D ^{1}H NMR spectra were recorded at oligonucleotide concentrations ranging from 0.6 to 1.1 mM, 100 mM LiCl concentration, pH 6.0 and 0 °C. Genes from where the sequences originate (annotated in Figure 10) are indicated next to individual spectrum.

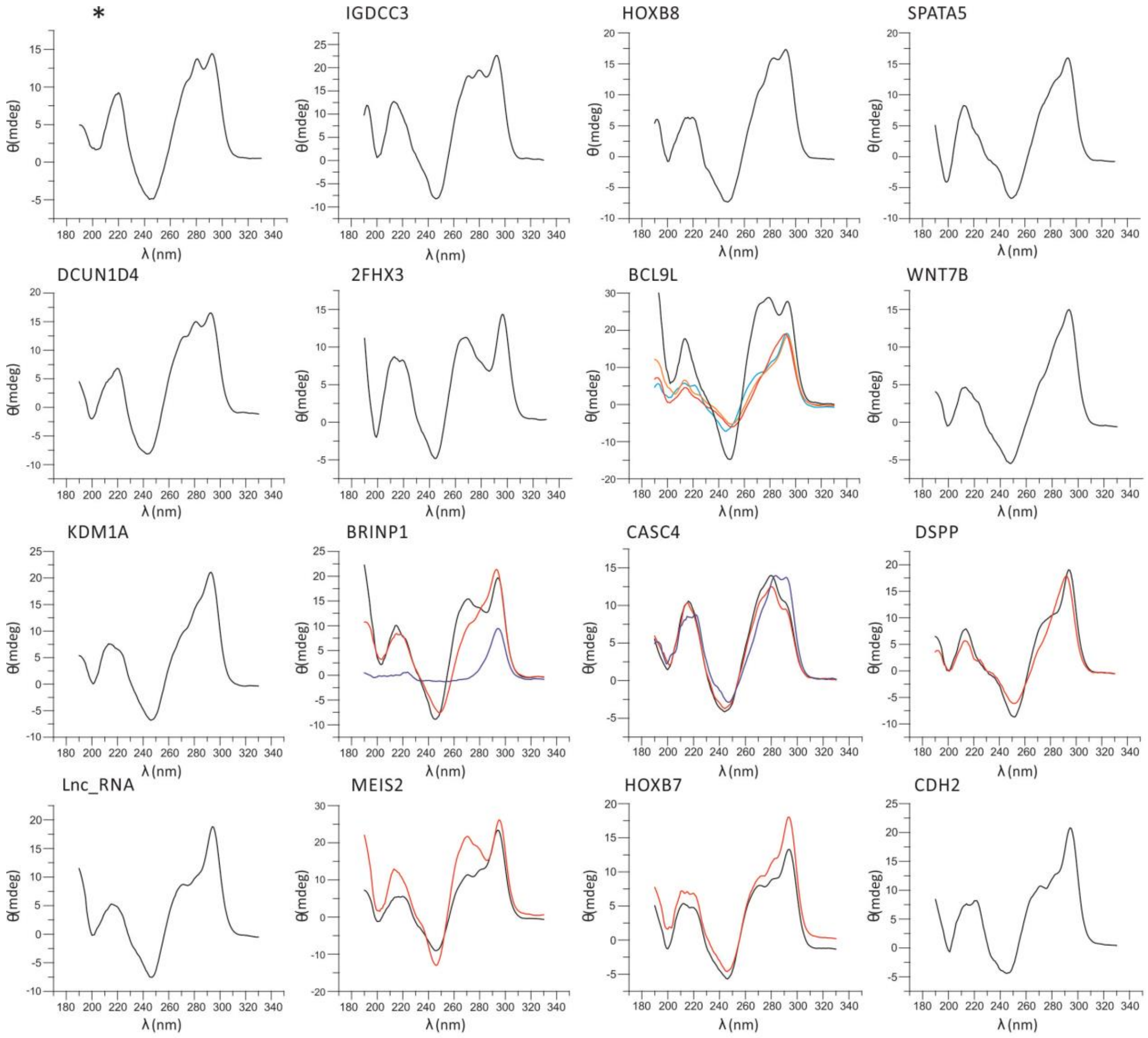

Supplementary Figure 26: CD spectra of oligonucleotides that contain AGCGA repeats found in different human genes. All CD spectra were recorded at concentrations close to 1.0 mM oligonucleotide concentrations in a 0.01 cm path-length cell at 100 mM LiCl, pH 6.0 and 0 °C. Genes (annotated in figure 6) from where the sequences originate are shown next to the CD spectra.

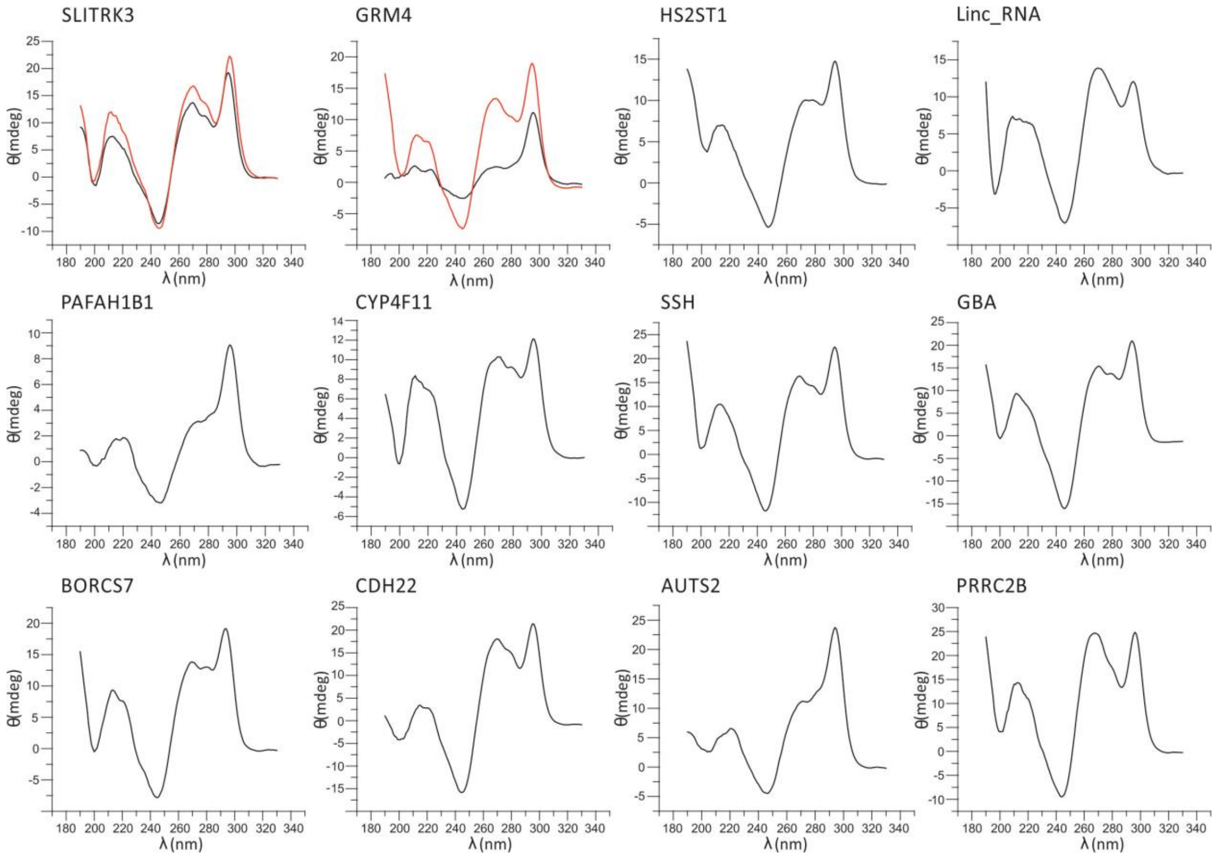

Supplementary Figure 27: CD spectra of oligonucleotides that contain AGCGA repeats found in different human genes. All CD spectra were recorded at concentrations close to 1.0 mM oligonucleotide concentrations in a 0.01 cm path-length cell at 100 mM LiCl, pH 6.0 and 0 °C. Genes (annotated in figure 6) from where the sequences originate are shown next to the CD spectra.

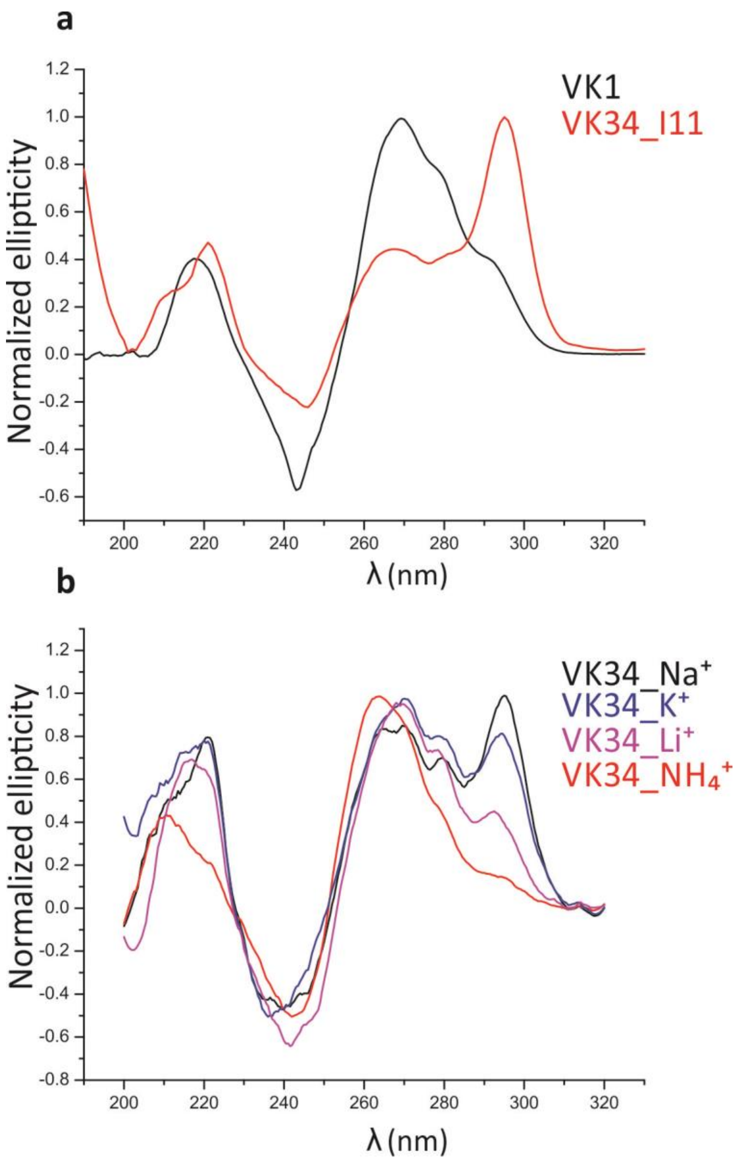

Supplementary Figure 28: CD spectra of VK1 and VK34_I11 oligonucleotides as well as VK34 in the presence of different cations. (a) VK1 and VK34_I11 oligonucleotides recorded in the presence of 100 mM LiCl. **(b)** CD spectra of VK34 recorded at around 1.0 mM oligonucleotide concentrations per strand, 100 mM salt solutions (NaCl, KCl, LiCl and NH_4Cl) in a 0.01 cm path-length cell at pH 6.0 and 0 °C.

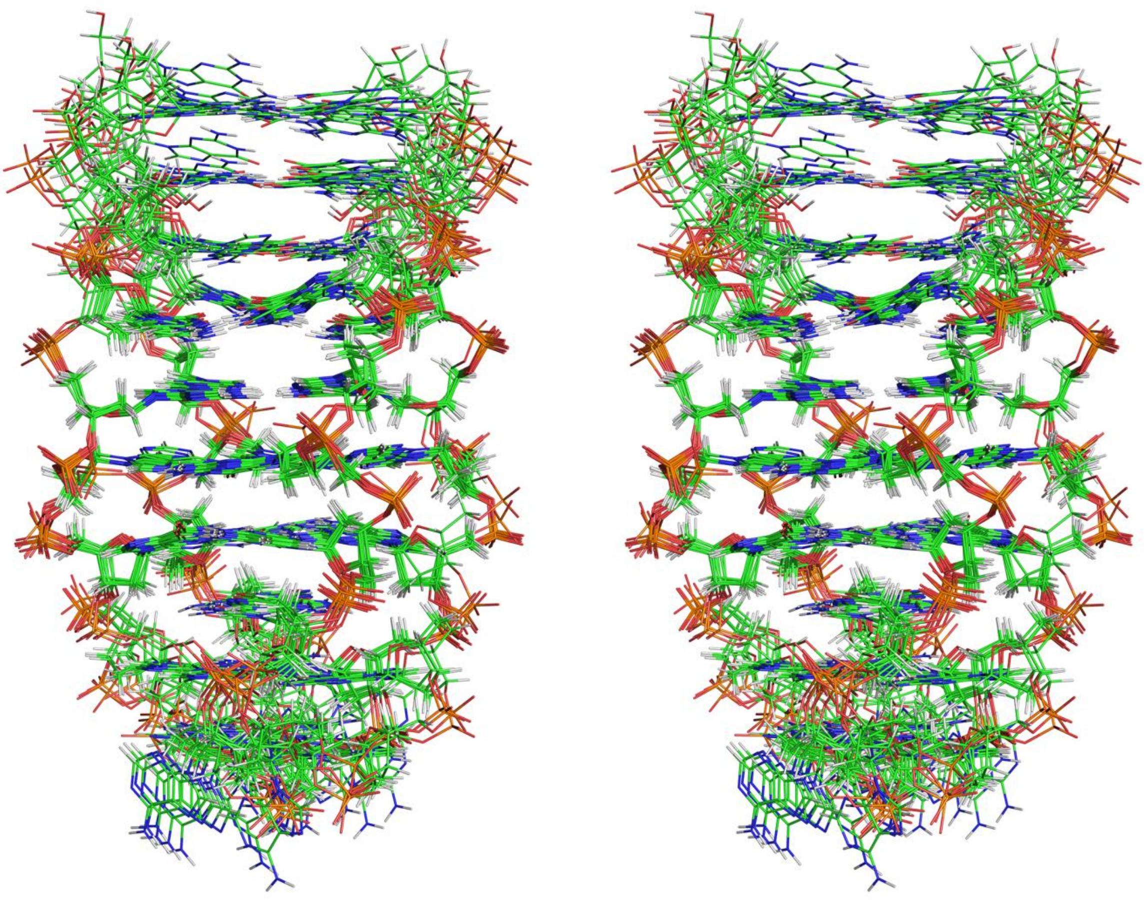

Supplementary Figure 29: Stereoscopic view of the 10 refined superimposed structures of VK34 dimer.

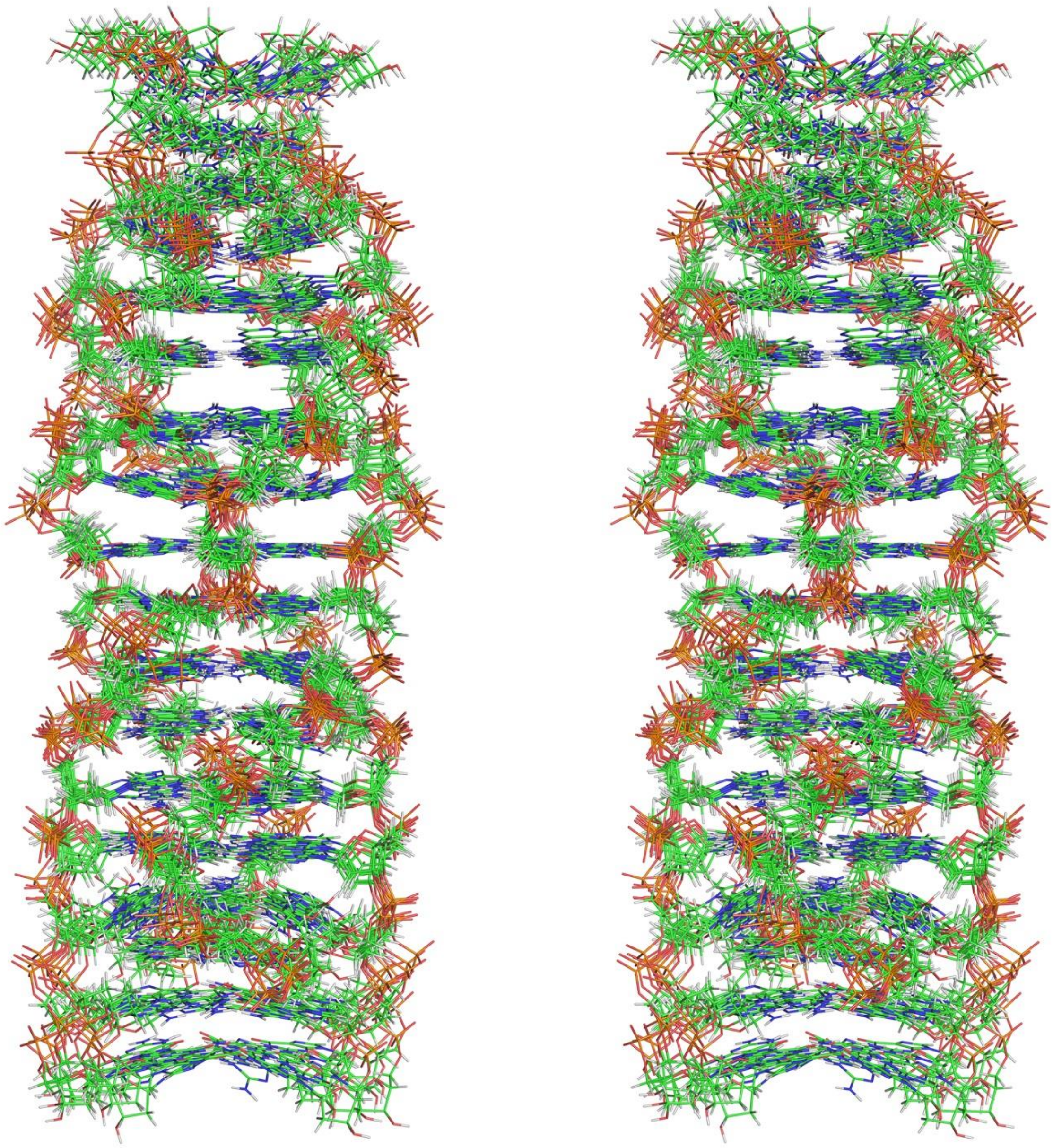

Supplementary Figure 30: Stereoscopic view of the 10 refined superimposed structures of VK34 tetramer.

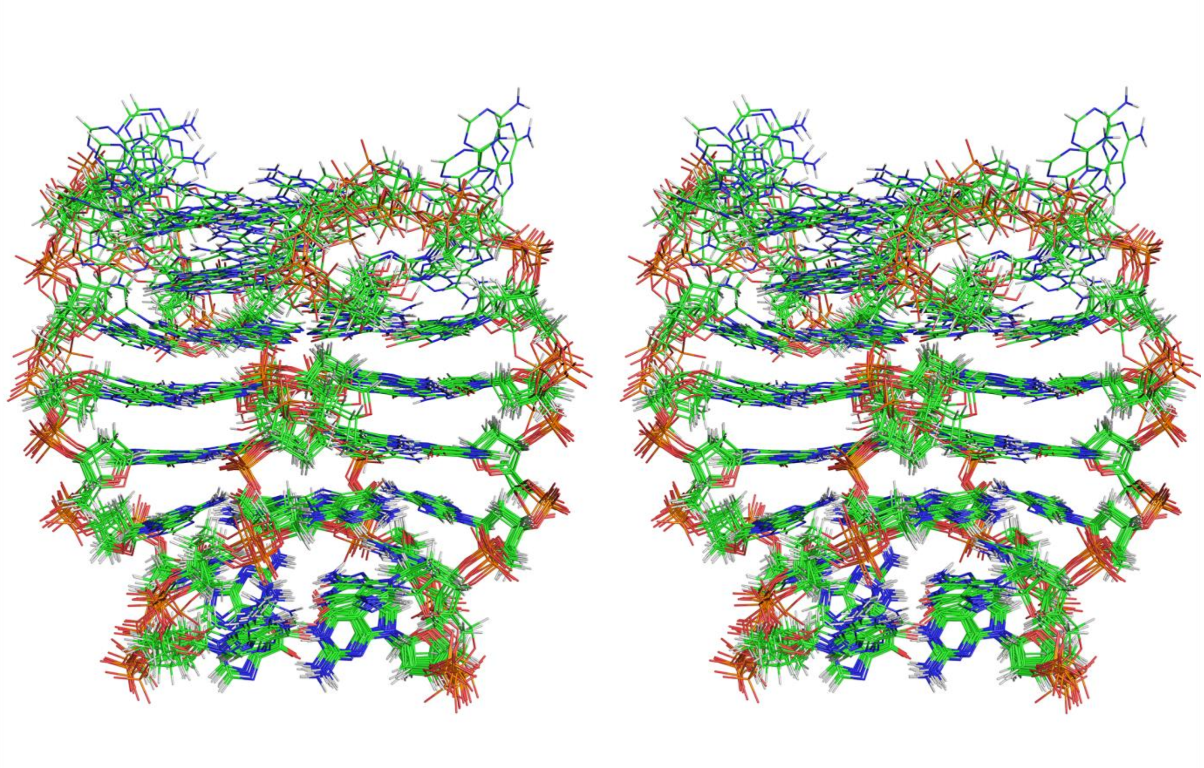

Supplementary Figure 31: Stereoscopic view of the 10 refined superimposed structures of VK34_I11.

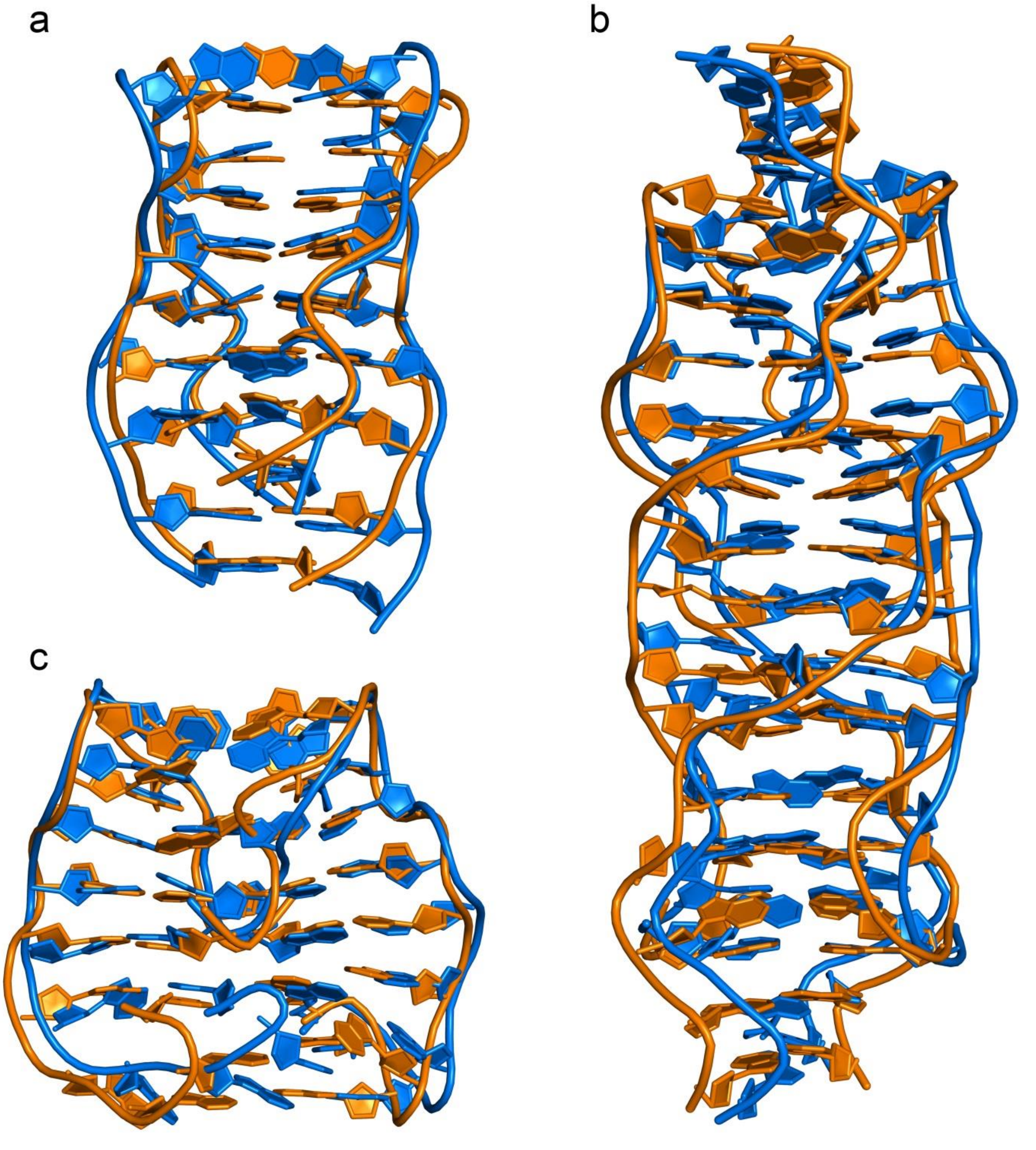

Supplementary Figure 32: Comparison of structures calculated with implicit water and planarity restraints (orange) and structures refined with explicit water model, randomly distributed cations and without planarity restraints (blue). (a) VK34 dimers. **(b)** VK34 tetramers. **(c)** VK34_I11 structures.

Supplementary Table 1: NMR restraints and structural statistics for VK34 dimer

	Non-exchangeable	Exchangeable
NOE-derived distance restraints		
Intranucleotide NOEs	194	0
Sequential (n, n+1)	128	22
Long-range (n, >n+1)	18	96
Torsion angle restraints	66	
Hydrogen bond restraints	24	
Structural statistics		
NOE violations >0.3 Å	0	
Deviations from idealized covalent geometry		
Bonds (Å)	0.0122 ± 0.0001	
Angles (deg)	2.38 ± 0.03	
Pairwise heavy-atom r.m.s.d. (Å)		
Overall	1.44 ± 0.39	
G3-A12-G3-A12 and G11-A4-G11-A4 quartets	0.48 ± 0.16	
G5-C10 residues	1.38 ± 0.62	
G1,C2 and G13-G15 residues	1.14 ± 0.31	

Supplementary Table 2: NMR restraints and structural statistics for VK34 tetramer

	Non-exchangeable	Exchangeable
NOE-derived distance restraints		
Intranucleotide NOEs	244	0
Sequential (n, n+1)	260	76
Long-range (n, >n+1)	44	164
Torsion angle restraints	144	
Hydrogen bond restraints	88	
Structural statistics		
NOE violations >0.3 Å	0	
Deviations from idealized covalent geometry		
Bonds (Å)	0.0127 ± 0.0001	
Angles (deg)	2.65 ± 0.02	
Pairwise heavy-atom r.m.s.d. (Å)		
Overall	1.54 ± 0.25	
Top part	1.09 ± 0.24	
Central part	1.21 ± 0.14	
Bottom part	1.05 ± 0.19	

Supplementary Table 3: NMR restraints and structural statistics for VK34_I11 structure

	Non-exchangeable	Exchangeable
NOE-derived distance restraints		
Intranucleotide NOEs	93	0
Sequential (n, n+1)	125	19
Long-range (n, >n+1)	16	25
Torsion angle restraints	60	
Hydrogen bond restraints	30	
Structural statistics		
NOE violations >0.3 Å	0	
Deviations from idealized covalent geometry		
Bonds (Å)	0.0124 ± 0.0001	
Angles (deg)	2.59 ± 0.02	
Pairwise heavy-atom r.m.s.d. (Å)		
Overall	1.39 ± 0.18	
Core of the structure; residues G1-G3 and A8-C10	0.77 ± 0.18	
Hydrophobic pocket; residues G3-A8	0.57 ± 0.14	
Without I11-G15 residues	0.71 ± 0.15	
Only I11-G15 residues	1.81 ± 0.29	

Supplementary Table 4: Phase angle of pseudorotation, chi, alpha, beta, gamma, delta, epsilon and zeta torsion angles* found in the family of ten structures of VK34 dimer.

	Phase angle (P)	Chi	Alpha	Beta	Gamma	Delta	Epsilon	Zeta
1. A	147 ± 35	271 ± 5	N. A.	N. A.	57 ± 2	128 ± 20	174 ± 5, 278 ± 7, 24 ± 7	276 ± 1, 106 ± 50
2. A	358 ± 1, 10 ± 5	251 ± 5	292 ± 3	173 ± 6	57 ± 4	92 ± 2	219 ± 3	300 ± 1
3. A	196 ± 5	198 ± 1	89 ± 1	174 ± 2	187 ± 1	158 ± 6	292 ± 2	73 ± 1
4. A	350 ± 11	199 ± 1	80 ± 1	203 ± 2	191 ± 8	96 ± 9	229 ± 5, 147 ± 1	308 ± 2, 44 ± 1
5. A	349 ± 79, 11 ± 9	233 ± 5	288 ± 2, 222 ± 1	175 ± 4	57 ± 1	105 ± 11	275 ± 5, 192 ± 1	185 ± 2, 277 ± 1
6. A	181 ± 62, 72 ± 1	258 ± 16	292 ± 6	173 ± 14	56 ± 7	137 ± 3, 85 ± 3	180 ± 11	274 ± 2
7. A	136 ± 19	241 ± 6	296 ± 2	166 ± 8	59 ± 2	129 ± 10	288 ± 7	169 ± 62, 83 ± 14
8. A	174 ± 14	210 ± 12	78 ± 3, 283 ± 13	244 ± 12, 126 ± 29	187 ± 8, 65 ± 12	149 ± 8	293 ± 14	93 ± 22
9. A	165 ± 13	264 ± 6	59 ± 3, 280 ± 11	175 ± 10	297 ± 4, 54 ± 4	149 ± 5	170 ± 4	272 ± 2
10. A	10 ± 3	228 ± 3	275 ± 5	181 ± 4	51 ± 2	87 ± 1	237 ± 9	310 ± 7
11. A	201 ± 7	200 ± 2	311 ± 1, 81 ± 1	170 ± 22	192 ± 1, 297 ± 1	158 ± 5	295 ± 5	76 ± 6
12. A	140 ± 16	244 ± 6	250 ± 19	157 ± 3	69 ± 5	127 ± 7	187 ± 5	279 ± 2
13. A	355 ± 18, 201 ± 10	252 ± 6	297 ± 5	174 ± 3	55 ± 3	110 ± 14	238 ± 25	206 ± 25
14. A	217 ± 7	265 ± 17	63 ± 11	176 ± 7	292 ± 3, 185 ± 11	153 ± 2	173 ± 9	276 ± 5
15. A	143 ± 5	251 ± 7	294 ± 2	171 ± 3	54 ± 3	133 ± 3	N. A.	N. A.
1. B	154 ± 18	274 ± 5	N. A.	N. A.	57 ± 2	136 ± 7	281 ± 4, 170 ± 5	156 ± 11, 276 ± 1
2. B	14 ± 7	246 ± 5	291 ± 3	118 ± 3, 172 ± 2	55 ± 4	90 ± 3	219 ± 3	298 ± 1
3. B	187 ± 7	198 ± 1	87 ± 3	179 ± 4	185 ± 3	156 ± 4	300 ± 12	75 ± 2
4. B	5 ± 2, 176 ± 1, 329 ± 1	201 ± 7	203 ± 27, 66 ± 3	201 ± 14	201 ± 23	100 ± 3	223 ± 9	308 ± 5
5. B	10 ± 9	230 ± 8	285 ± 3	174 ± 3	56 ± 4	98 ± 10	191 ± 3, 276 ± 2	275 ± 7, 185 ± 1
6. B	74 ± 3, 183 ± 16	248 ± 18	297 ± 6	180 ± 10	61 ± 7	99 ± 21	182 ± 10	274 ± 1
7. B	146 ± 13	245 ± 5	295 ± 2	167 ± 7	58 ± 1	133 ± 9	290 ± 8	103 ± 28
8. B	176 ± 15	207 ± 14	272 ± 10, 76 ± 4	256 ± 5, 116 ± 39	182 ± 14, 66 ± 5	148 ± 5	290 ± 11	83 ± 6, 127 ± 8
9. B	167 ± 12	266 ± 8	286 ± 1, 62 ± 4	182 ± 12	299 ± 5, 50 ± 1	147 ± 8	171 ± 4	271 ± 3
10. B	8 ± 2	230 ± 2	278 ± 3	178 ± 5	52 ± 2	89 ± 1	248 ± 1	318 ± 5
11. B	205 ± 5	201 ± 2	312 ± 1	200 ± 3	299 ±2	159 ± 5	292 ± 6	71 ± 4
12. B	153 ± 7	253 ± 6	245 ± 3, 76 ± 1	178 ± 21	64 ± 5, 192 ± 1	134 ± 7	177 ± 7	274 ± 1
13. B	314 ± 41	257 ± 5	295 ± 4	183 ± 3	57 ± 3	106 ± 8	212 ± 19	220 ± 23
14. B	232 ± 43	270 ± 7	66 ± 8	180 ± 9	296 ± 3	151 ± 1	172 ± 5	274 ± 1
15. B	143 ± 3	252 ± 7	294 ± 1	172 ± 8	54 ± 1	134 ± 2	N. A.	N. A.

In degrees.*

Supplementary Table 5: Phase angle of pseudorotation, chi, alpha, beta, gamma, delta, epsilon and zeta torsion angles* found in the family of ten structures of VK34 tetramer.

	Phase angle (P)	Chi	Alpha	Beta	Gamma	Delta	Epsilon	Zeta
1. A	162 ± 17	255 ± 27	N. A.	N. A.	53 ± 1,<br>298 ± 1,<br>185 ± 1	144 ± 13	134 ± 43,<br>311 ± 9	278 ± 10,<br>92 ± 15
2. A	136 ± 28	176 ± 3<br>190 ± 8	292 ± 8,<br>208 ± 4,<br>65 ± 1	185 ± 2,<br>177 ± 3	53 ± 4,<br>304 ± 2,<br>187 ± 4	122 ± 19	14 ± 7,<br>135 ± 9	60 ± 6,<br>290 ± 4
3. A	147 ± 11	179 ± 1	214 ± 6,<br>289 ± 1,<br>72 ± 2	196 ± 6,<br>164 ± 6	48 ± 3,<br>197 ± 6	134 ± 10	309 ± 19	78 ± 4
4. A	135 ± 31	210 ± 10	76 ± 2,<br>224 ± 18	216 ± 5,<br>131 ± 40	207 ± 4,<br>175 ± 1,<br>71 ± 3	120 ± 22	205 ± 15	283 ± 6
5. A	123 ± 17	246 ± 14	287 ± 11	161 ± 34	55 ± 4	115 ± 15	33 ± 8,<br>255 ± 39,<br>177 ± 2	59 ± 1,<br>168 ± 7,<br>273 ± 4
6. A	125 ± 12	218 ± 6	295 ± 6	154 ± 22	61 ± 4	119 ± 11	214 ± 11	293 ± 2
7. A	173 ± 17	179 ± 1	290 ± 7,<br>193 ± 3	192 ± 7,<br>167 ± 16	314 ± 5,<br>52 ± 1	151 ± 11	318 ± 23	76 ± 3
8. A	332 ± 11,<br>8 ± 1,<br>216 ± 212	197 ± 5	78 ± 3,<br>192 ± 4	198 ± 6<br>114 ± 33	196 ± 7,<br>162 ± 4	102 ± 13	261 ± 35	304 ± 11
9. A	140 ± 15	65 ± 13	280 ± 13,<br>79 ± 17	159 ± 11,<br>57 ± 2,<br>193 ± 1	59 ± 6,<br>188 ± 7,<br>177 ± 1	127 ± 12	169 ± 3,<br>184 ± 2,<br>43 ± 18	279 ± 2,<br>58 ± 3
10. A	134 ± 9	204 ± 12	298 ± 5,<br>125 ± 35	165 ± 2,<br>190 ± 4	64 ± 5,<br>179 ± 1,<br>187 ± 6	128 ± 11	222 ± 16	298 ± 4
11. A	167 ± 11	179 ± 1	159 ± 27,<br>302 ± 3	240 ± 7,<br>170 ± 2	52 ± 2,<br>317 ± 3	149 ± 8	297 ± 7	85 ± 4
12. A	152 ± 4	237 ± 8	79 ± 1,<br>242 ± 5	197 ± 12,<br>164 ± 5	194 ± 1,<br>65 ± 2	138 ± 2	221 ± 12	304 ± 5
13. A	155 ± 17	202 ± 2	197 ± 5,<br>303 ± 1	190 ± 4,<br>174 ± 1	59 ±1,<br>323 ± 4	139 ± 10	297 ± 11	104 ±16
14. A	144 ± 11	270 ± 9	42 ± 2,<br>256 ± 10	164 ± 8,<br>185 ± 2	275 ± 2,<br>57 ± 4	134 ± 9	209 ± 9	288 ± 6
15. A	191 ± 15	212 ± 8	189 ± 4,<br>296 ± 2	182 ± 1,<br>173 ± 3	302 ± 1,<br>56 ± 2	147 ± 7	N. A.	N. A.
1. B	157 ± 18	258 ± 12	N. A.	N. A.	54 ± 1,<br>298 ± 2	141 ± 13	308 ± 5,<br>109 ± 47	92 ± 12,<br>287 ± 16
2. B	141 ± 13	174 ± 3<br>188 ± 4	68 ± 3,<br>265 ± 37	186 ± 4,<br>176 ± 5	195 ± 6,<br>53 ± 5,<br>305 ± 1	127 ± 8	334 ± 15,<br>147 ± 22	265 ± 24,<br>88 ± 17
3. B	172 ± 18	179 ± 1	63 ± 11,<br>292 ± 10	169 ± 8,<br>189 ± 3	298 ± 10,<br>200 ±2,<br>47 ± 1	152 ± 12	328 ± 17	75 ± 6
4. B	152 ± 23	233 ± 13	229 ± 22,<br>59 ± 11	189 ± 8,<br>126 ± 37	62 ± 2,<br>240 ± 20,<br>169 ± 5	130 ± 19	202 ± 28,<br>123 ± 57	284 ± 10
5. B	120 ± 8	238 ± 16	281 ± 28,<br>121 ± 4	173 ± 2,<br>188 ± 3	54 ± 5,<br>189 ± 6	110 ± 9	173 ± 5,<br>223 ± 42	272 ± 4

6. B	136 ± 6	220 ± 6	296 ± 7	164 ± 18	61 ± 4	128 ± 5	206 ± 7	290 ± 2
7. B	162 ± 12	179 ± 1	195 ± 6, 295 ± 3	196 ± 9	52 ± 1, 318 ± 2	144 ± 11	313 ± 22	76 ± 3
8. B	212 ± 14	205 ± 12	207 ± 14, 64 ± 16	197 ± 4, 98 ± 23	229 ± 28, 171 ± 3	140 ± 20	229 ± 21	293 ± 7
9. B	131 ± 18	78 ± 12	282 ± 11, 92 ± 1	138 ± 39, 198 ± 8	52 ± 7, 195 ± 1	119 ± 16	170 ± 5, 34 ± 4	55 ± 2, 278 ± 3
10. B	127 ± 15	201 ± 10	90 ± 1, 291 ± 22	188 ± 1, 170 ± 4	202 ±2, 69 ± 7	122 ± 15	228 ± 18, 57 ± 2	291 ± 2, 134 ± 1
11. B	177 ± 13	179 ± 1	175 ± 2, 298 ± 9	232 ± 3, 157 ± 21	55 ± 2, 315 ±7	157 ± 11	318 ± 9	87 ± 5
12. B	148 ± 5	241 ± 3	240 ± 2	172 ± 4, 181 ± 1	61 ± 3	136 ± 4	217 ± 10	304 ± 7
13. B	155 ± 13	204 ± 4	201 ± 13, 84 ± 1	177 ± 1, 190 ± 3	57 ± 1, 174 ± 1	141 ± 8	280 ± 8	159 ± 22
14. B	180 ± 12	203 ± 3	294 ± 2	167 ± 5	306 ± 3	159 ± 7	288 ± 5	94 ± 33
15. B	137 ± 37	224 ± 17	80 ± 2, 267 ± 17	249 ± 31, 93 ± 50	193 ± 2, 172 ± 1	123 ± 20	N. A.	N. A.
1. C	159 ± 10	264 ± 10	N. A.	N. A.	298 ± 1, 55 ± 2	149 ± 8	316 ± 8	81 ± 2
2. C	125 ± 9	173 ± 2 193 ± 4	211 ± 11, 70 ± 3	189 ± 4	55 ± 2, 186 ± 2	118 ± 7	176 ± 2, 21 ± 13, 330 ± 15	238 ± 3, 60 ± 10, 115 ± 2
3. C	160 ± 16	179 ± 1	58 ± 14, 219 ± 3, 322 ± 2	190 ± 7, 166 ± 4	293 ± 5, 49 ± 1, 202 ± 1	140 ± 11	299 ± 8, 73 ± 1	81 ± 7, 297 ± 1
4. C	155 ± 13	232 ± 5	73 ± 2, 258 ± 23	203 ± 2, 167 ± 8	207 ± 2, 72 ± 6	135 ± 10	201 ± 18	274 ± 15
5. C	134 ± 18	248 ± 10	283 ± 18	174 ± 5, 183 ± 1, 64 ± 1	55, ± 3 177 ± 1, 183 ± 1	121 ± 16	172 ± 6, 194 ± 9	274 ± 6
6. C	130 ± 13	219 ± 10	294 ± 11	168 ± 3	61 ± 3, 192 ± 1, 177 ± 1	122 ± 12	216 ± 11	292 ± 7
7. C	175 ± 16	179 ± 1	297 ± 3, 179 ± 38, 196 ± 9	179 ± 1, 195 ± 7	317 ±2, 58 ±7	142 ± 8	67 ± 2, 314 ± 17	304 ± 2, 77 ± 1
8. C	216 ± 17, 348 ± 2, 12 ± 2	200 ± 6	198 ± 8, 110 ± 43	172 ± 3, 188 ± 7	188 ± 7, 173 ± 1	119 ± 29	235 ± 24, 90 ± 5	293 ± 8, 74 ± 4
9. C	133 ± 13	79 ± 11	277 ± 21, 82 ± 12	144 ± 37, 200 ± 8	51 ± 4, 194 ± 2, 175 ± 3	120 ± 13	160 ± 20, 56 ± 10	281 ± 10, 60 ± 6
10. C	136 ± 6	205 ± 9	289 ± 29, 129 ± 43	170 ± 2, 192 ± 4	68 ± 4, 175 ± 1, 194 ± 1	132 ± 6	238 ± 14	297 ± 5
11. C	177 ± 9	179 ± 1	303 ± 2, 171 ± 4	171 ± 3, 218 ± 26	316 ± 1, 47 ± 4	156 ± 11	316 ± 8	84 ±2
12. C	151 ± 9	240 ± 4	240 ± 4	172 ± 6	67 ± 7	136 ± 4	220 ± 12	304 ± 5
13. C	154 ± 16	203 ± 3	197 ± 5	188 ± 3, 175 ± 2	59 ± 1 , 323 ± 1	139 ± 12	300 ± 8	96 ± 13
14. C	150 ± 14	272 ± 12	40 ± 3, 253 ± 4	170 ± 6	279 ± 2, 58 ± 4	137 ± 10	211 ± 7	288 ± 6

15. C	190 ± 14	214 ± 10	191 ± 3	175 ± 3, 184 ± 4	56 ± 2	146 ± 4	N. A.	N. A.
1. D	162 ± 20	241 ± 30	N. A.	N. A.	45 ± 2, 298 ± 1, 180 ±1,	144 ± 9	296 ± 44, 169 ± 6	259 ± 8, 97 ± 30
2. D	130 ± 15	190 ± 8 172 ± 2	285 ± 14, 210 ± 8	184 ± 2, 173 ± 2	50 ± 5, 305 ± 1, 189 ±1	114 ± 10	358 ± 15, 146 ± 22	68 ± 7, 271 ±22
3. D	156 ± 14	179 ± 1	211 ± 5, 61 ± 13, 289 ± 2	190 ± 5, 166 ± 9	52 ±2, 194 ± 1, 289 ±1	140 ± 14	65 ± 1, 317 ± 12	305 ± 1, 75 ± 3
4. D	154 ± 33	229 ± 10	214 ± 24, 58 ± 14	192 ± 12	225 ± 33, 64 ± 4, 178 ± 1	135 ± 19	205 ± 16, 119 ± 47	286 ± 6
5. D	114 ± 17	240 ± 19	287 ± 7, 108 ± 39	158 ± 35, 188 ± 3	52 ± 3, 190 ±8, 168 ± 1	107 ± 15	174 ± 3, 226 ± 31, 24 ± 2	262 ± 32, 59 ± 2
6. D	137 ± 9	211 ± 11	288 ± 13, 83 ± 5	149 ± 44, 207 ± 31	59 ± 3, 191 ± 2, 177 ±1	131 ± 5	215 ± 9	298 ± 6
7. D	170 ± 14	179 ± 1	291 ± 3, 192 ± 8	194 ± 10, 175 ± 5	314 ± 2, 53 ± 3	149 ± 8	298 ± 20	76 ± 4
8. D	5 ± 2, 197 ± 12	209 ± 4	79 ± 6, 202 ± 1	205 ± 6, 88 ± 1	196 ± 7, 169 ± 2	130 ± 33	82 ± 2, 217 ± 9	289 ± 4, 71 ± 1
9. D	129 ± 17	75 ± 6	271 ± 26, 85 ± 10	165 ± 4, 195 ± 3	52 ± 8, 190 ± 3	117 ± 15	164 ± 11, 42 ± 8	279 ± 5, 54 ± 4
10. D	133 ± 11	197 ± 3	300 ± 4, 88 ± 6	169 ± 68, 189 ± 2	69 ± 6, 194 ± 5	127 ± 14	229 ± 19	292 ± 4
11. D	179 ± 13	179 ± 1	156 ± 35, 302 ± 1	233 ± 3, 17 ± 1	60 ± 5, 322 ± 1	156 ± 11	309 ± 6	86 ±3
12. D	154 ± 2	233 ± 4	234 ± 1	178 ± 1, 185 ± 4	61 ± 3	139 ± 3	215 ± 3	308 ± 5
13. D	150 ± 6	202 ± 2	199 ± 9, 86 ± 2	190 ± 6, 179 ± 1	58 ± 1, 174 ± 3	139 ± 6	278 ± 2	165 ± 8
14. D	180 ± 10	202 ± 1	295 ± 2	172 ± 5	305 ± 3	158 ± 5	287 ± 8	78 ± 4, 159 ±3
15. D	137 ± 35	224 ± 18	80 ± 3, 268 ± 17	249 ± 30, 55 ± 47	194 ± 3, 147 ± 43	120 ± 17	N. A.	N. A.

In degrees.*

Supplementary Table 6: RMSD values of VK34 dimer structures refined in explicit water model with randomly distributed cations and without planarity restraints in comparison to the lowest energy structure calculated with implicit water and planarity restraints.

Structure ID	Overall	G3-A12-G3-A12 and G11-A4-G11-A4 quartets	G5-C10 residues	G1,C2 and G13-G15 residues
1	2.0	1.3	1.1	1.9
2	2.1	1.5	1.0	1.8
3	1.6	1.2	0.8	1.4
4	1.7	1.4	0.9	1.5
5	1.8	1.4	1.3	1.7
6	1.8	1.2	1.1	1.9
7	1.3	1.0	1.2	1.2
8	2.0	1.2	0.8	2.3
9	2.3	1.0	2.0	2.0
10	1.5	1.0	1.0	1.5
AVERAGE	1.8	1.2	1.1	1.7

Supplementary Table 7: RMSD values of VK34 tetramer structures refined in explicit water model with randomly distributed cations and without planarity restraints in comparison to the lowest energy structure of VK34 tetramer calculated with implicit water and planarity restraints.

Structure ID	Overall	Central part	Top part	Bottom part
1	1.8	1.1	1.5	1.3
2	1.8	1.3	1.6	1.8
3	2.5	1.5	1.6	1.5
4	1.5	1.4	1.0	1.6
5	1.8	1.0	1.7	1.2
6	1.9	1.4	1.3	1.4
7	1.7	1.4	1.0	1.0
8	2.5	1.4	1.4	1.4
9	1.9	1.3	1.6	1.5
10	1.5	1.3	0.8	1.7
AVERAGE	1.9	1.3	1.4	1.4

Supplementary Table 8: RMSD values of VK34_I11 structures refined in explicit water model with randomly distributed cations and without planarity restraints in comparison to the lowest energy structure of VK34_I11 calculated with implicit water and planarity restraints.

Structure ID	Overall	Core of the structure; residues G1-G3 and A8-C10	Hydrophobic pocket; residues G3-A8	Without I11-G15 residues	Only I11-G15 residues
1	2.1	1.1	1.1	1.1	3.0
2	1.8	1.0	1.2	1.2	1.8
3	1.8	1.0	1.2	1.1	1.7
4	1.7	1.0	1.2	1.1	1.7
5	2.1	1.2	0.9	1.1	2.0
6	1.8	0.7	1.0	1.0	2.5
7	1.8	1.3	1.1	1.3	1.8
8	2.7	0.9	1.0	1.1	3.0
9	1.6	1.1	1.1	1.2	1.6
10	2.0	1.2	1.3	1.3	2.0
AVERAGE	1.9	1.1	1.1	1.15	2.10
